# Willingness to accept COVID-19 vaccination among people living with HIV in a high HIV prevalence community

**DOI:** 10.1186/s12889-022-13623-w

**Published:** 2022-06-22

**Authors:** Sabina Govere-Hwenje, Jana Jarolimova, Joyce Yan, Anele Khumalo, Gugulami Zondi, Marcia Ngcobo, Nafisa J. Wara, Dani Zionts, Laura M. Bogart, Robert A. Parker, Ingrid V. Bassett

**Affiliations:** 1 AIDS Healthcare Foundation, Durban, South Africa; 2 Massachusetts General Hospital, Division of Infectious Diseases, Boston, USA; 3 Massachusetts General Hospital, Medical Practice Evaluation Center, Boston, USA; 4 Massachusetts General Hospital, Biostatistics Center, Boston, USA; 5 RAND Corporation, Santa Monica, USA; 6 Harvard University, Center for AIDS Research (CFAR), Boston, USA; 7 Harvard Medical School, Boston, USA

**Keywords:** Vaccination, vaccine hesitancy, COVID-19, HIV, South Africa

## Abstract

**Background:**

People living with HIV (PLWH) may have a poorer prognosis with COVID-19 infection and are an important population for COVID-19 vaccination. We assessed the willingness and reasons for COVID-19 vaccine acceptance or hesitancy among PLWH in South Africa.

**Methods:**

We conducted a cross-sectional study consisting of telephone interviews with a randomly selected subset of participants enrolled in a prospective observational cohort study evaluating a decentralized antiretroviral therapy (ART) delivery program in South Africa. Questions assessed willingness to accept a future COVID-19 vaccine, concerns regarding COVID-19 vaccination, and overall vaccine confidence. Interviews were conducted between September 2020 and January 2021. We evaluated participant demographics, sources of COVID-19 information, stigma and medical mistrust, uptake of non-pharmaceutical interventions, and socioeconomic impacts of the COVID-19 pandemic as potential covariates of willingness to accept vaccination.

**Results:**

We completed interviews with 213 participants; 153 (72%) were female, median age 35y, and 100 (47%) had completed secondary school. Among the participants, 121 (57%) were willing to accept future vaccination, 46 (22%) were unsure, and 45 (21%) stated they did not intend to be vaccinated. Fear of side effects, reported by 42 (20%), was the most common concern about COVID-19 vaccination. Older age was associated with willingness to accept vaccination (aOR 1.75 for every 10-year increase in age, 95% CI 1.10–2.78, *p* = 0.02), while higher medical mistrust related to COVID-19 (aOR 0.21, 95% CI 0.093–0.45, *p* < 0.001) and use of social media for COVID-19 information (aOR 0.30, 95% CI 0.11–0.84, *p* = 0.02) were associated with lower willingness to accept vaccination.

**Conclusions:**

In this cohort of PLWH in South Africa, over half were willing to accept COVID-19 vaccination, although a substantial proportion remained unsure or were not willing to be vaccinated. Public health messaging should emphasize the safety and efficacy of COVID-19 vaccination and address misinformation and medical mistrust among PLWH. Ongoing efforts to ensure access to COVID-19 vaccines for vulnerable populations are crucial.

**Supplementary Information:**

The online version contains supplementary material available at 10.1186/s12889-022-13623-w.

## Background

The novel coronavirus disease 2019 (COVID-19) pandemic has created an unprecedented global health challenge [1, 2]. Development and global rollout of effective COVID-19 vaccines is a crucial step in controlling the pandemic, alongside non-pharmaceutical interventions, diagnostic capacity, and treatment research [3]. Vaccination is considered to be one of the greatest historical successes of public health and has contributed significantly to the global decline in morbidity and mortality from other infectious diseases [4]. Vaccination programs depend on high and persistent vaccine uptake to reduce the incidence of vaccine-preventable diseases such as COVID-19 [5].

While the first COVID-19 vaccine candidates showing efficacy in clinical trials have been rolled out worldwide, significant concern remains about vaccine supply, delivery, and uptake in low- and middle-income countries. The World Health Organization (WHO) Strategic Advisory Group of Experts (SAGE) Working Group on Vaccine Hesitancy defines “vaccine hesitancy” as a “delay in acceptance or refusal of vaccines despite availability of vaccine services” [6]. Vaccine hesitancy had already been identified by the WHO as one of the ten most important threats to global health in 2019 [7], and now could significantly limit the efficacy of a COVID-19 vaccine if an inadequate portion of the global population is immunized.

Vaccine hesitancy differs across time, place, and specific vaccines being offered [4]. Understanding rates of and reasons for hesitancy and predictors of readiness among specific populations continues to be crucial to inform scientific decisions on COVID-19 vaccine uptake [8]. People living with HIV (PLWH) worldwide form a vulnerable population who may be at risk for worse clinical outcomes from COVID-19 [9] and for whom COVID-19 vaccination may carry a particular benefit. However, vaccine hesitancy, related to either current or future COVID-19 vaccines, in this population is not currently well understood. In South Africa, it is estimated that 8.2 million people are living with HIV, representing 13.7% of the national population, with an HIV prevalence of 19.5% among those ages 15–49 [10]. Thus, understanding willingness to accept COVID-19 vaccination in this population will have significant impact on approaches to national vaccine distribution. While COVID-19 vaccination had not yet been introduced in South Africa at the time of data collection, current COVID-19 vaccine uptake as of March 2022 remains below target [11] and efforts to understand vaccine acceptance and hesitancy among priority populations in South Africa are ongoing. We aimed to measure willingness to accept vaccination against COVID-19 before vaccine rollout among PLWH on antiretroviral therapy (ART) and to identify factors associated with willingness to accept vaccination against COVID-19.

## Methods

### Study design

This was a cross-sectional study conducted through telephone interviews between 25 September 2020 and 8 January 2021, prior to the availability of COVID-19 vaccines in South Africa.

### Study population

We contacted a subset of participants enrolled in a prospective observational cohort study evaluating a decentralized ART delivery program in KwaZulu-Natal province, South Africa. The parent study recruited participants meeting eligibility criteria for the national Chronic Medicine Dispending and Distribution (CCMDD) program (i.e., not pregnant, on ART for ≥1 year, and virologically suppressed as per program guidelines) in nine public sector clinics offering the CCMDD program in the urban township of Umlazi [12]. At enrollment into the parent study, participants completed a baseline questionnaire assessing demographic characteristics, HIV care history, barriers to HIV care, competing needs, mental health, and social support, and agreed to be contacted later by telephone. Competing needs assessed whether in the preceding 6 months, the participant had gone without healthcare because they needed the money for basic needs, such as food, clothing, or housing, or if they had gone without basic needs because they needed the money for healthcare [13]. Baseline data were collected between October 2018 and March 2020.

For this study, we contacted a random subsample of the 2220 participants enrolled in the parent study [14]. 900 participants were chosen using random sampling, stratified to include equal numbers of participants enrolled in the decentralized ART delivery program for 0–6 months, 6–12 months, and > 12 months, and equal proportions from each clinic site. Questions pertaining to COVID-19 vaccination were added partway through the process of telephone interviews for this randomly selected subsample and completed for all subsequent respondents (Supplementary File 1). We did not calculate an optimal sample size; the number of participants included in the study was dictated by staff availability and response rate. Three trained, bilingual research assistants telephoned participants at the number provided at parent study enrolment. Participants who provided verbal informed consent were administered a semi-structured questionnaire by the research assistant in their preferred language (isiZulu or English), with each interview lasting 25–30 minutes.

### Data collection

#### Willingness to accept COVID-19 vaccination and overall vaccine confidence

To evaluate levels of acceptance of a future COVID-19 vaccine, we asked participants, “do you intend to accept future COVID-19 vaccination for yourself?” We asked participants to list all concerns regarding a future COVID-19 vaccine. For further context, we assessed overall vaccine confidence, defined as the “belief that vaccination serves the best health interests of the public and its constituents” [15] along with trust in vaccines, vaccine providers, and vaccine decision-makers [16]. To assess vaccine confidence, we asked for level of agreement or disagreement with four statements evaluating importance, safety, effectiveness, and religious compatibility of vaccination, adapted from a recent worldwide study of vaccine confidence [17]. To ascertain underlying local rates of adult vaccination in this population, we asked whether participants had been vaccinated against seasonal influenza in 2019, and reasons for not doing so for those who did not. In order to assess the internal consistency of our question on willingness to accept vaccination, we created a summary COVID-19 vaccine confidence measure, assigning one point for those reporting no concerns regarding COVID-19 vaccination, and one additional point for disagreement with each of the following statements: “if a vaccine were available to prevent COVID-19 in the future, I: ‘would not want to get it’, ‘would not trust it’, or ‘am worried that it could be harmful’”. These items were adapted from a previous study of COVID-19 medical mistrust and vaccine hesitancy among PLWH [18]. The measure had a possible score range of 0–4, with higher scores indicating greater COVID-19 vaccine confidence.

#### HIV care history, reactions to COVID-19, stigma, and medical mistrust

Demographic data, HIV care history, and measures of healthcare access were obtained from the baseline questionnaire of the parent study (Supplementary File 2). In the COVID-19 telephone interview, we asked participants about their sources of information on COVID-19 and changes in daily activities due to the pandemic (all that apply). We defined ‘recommended’ changes in daily activities as those falling within the recommendations of the National Department of Health of South Africa [19]. The activities include physical distancing (avoiding large gatherings, not hugging other people, avoiding people who present with symptoms, avoiding public transport, not going outside), mask wearing, and hygiene (washing hands, not touching face, using hand sanitizer frequently). We assessed stigma related to COVID-19 using six questions adapted from previously published stigma scales for HIV and chronic illness [20–22] and described in detail previously [12]. We assessed medical mistrust related to COVID-19 using seven questions; two adapted from a published scale assessing conspiracy theories around HIV [23] and the remainder developed for the current study, as described previously [12]. We defined medical mistrust as distrust in healthcare systems and medical providers with the belief that they are acting against one’s best interest [24–26]. Responses to stigma and medical mistrust questions were on a 5-point Likert scale with scores ranging 0–4, with higher scores indicating higher stigma or medical mistrust. We calculated summary scores for overall COVID-19 stigma and medical mistrust by adding the scores for each individual question.

#### Statistical analysis

We used descriptive statistics (median, interquartile range [IQR], frequency) to report baseline and COVID-era participant characteristics, sources of information on COVID-19, levels of COVID-19 stigma and medical mistrust, and responses to questions on COVID-19 vaccination and general vaccine confidence. For modeling measures of medical mistrust and stigma related to COVID-19, we categorized data into above and below the median to use the two variables consistently in the analysis. This also allows for easy interpretation of the effect of the variables. We determined internal consistency among the questions on COVID-19 vaccine confidence using Cronbach’s alpha. A Cochran-Armitage test of trend was used to assess the relationship of willingness to accept vaccination with the summary COVID-19 vaccine confidence measure. We used univariate and multivariable logistic regression to assess predictors of willingness to accept COVID-19 vaccine (as defined by an answer of “yes” to “do you intend to accept future COVID-19 vaccination for yourself?”). Factors with *p* < 0.05 in univariate logistic regression models were included in a multivariable model in addition to age and sex, which were pre-specified. All reported *p*-values were two-tailed, and *p* < 0.05 was considered statistically significant. Analyses were conducted using SAS software (version 9.4, SAS Institute, Cary, NC).

#### Ethical considerations

The study protocol was approved by the Biomedical Research Ethics Council of the University of KwaZulu-Natal (Protocol BE092/18) and by the Partners Healthcare Institutional Review Board (Protocol 2017P001690).

## Results

### Participant characteristics

Two hundred and thirteen participants consented to and completed the interview. Among the participants, 153 (72%) were female, 212 (99.5%) identified their ethnicity as Black, median age was 35 (interquartile range [IQR], 29–43), and 100 (47%) had completed secondary school. Median time since ART initiation was 2.0 years (IQR 1.0–4.0). The minority (82, 39%) were employed at enrolment, all (100%) reported good, very good, or excellent ability to take HIV medication, and only 27 participants (13%) reported any competing needs at baseline (Table 1).

**Table 1:** Participant characteristics, sources of information, and responses to COVID-19; *n* = 213 unless otherwise specified

Variable	Median, [IQR] or n, (%)
Gender	
Female	153 (72)
Male	60 (28)
Ethnicity	
Black	212 (99.5)
White	1 (0.5)
Age, years	35 [29–43]
Education level at baseline^a^	
Primary school or less	13 (6)
Some high school	100 (47)
Matric	84 (39)
Tertiary	16 (8)
Employed at baseline	82 (39)
Ability to take HIV medication	
Very poor, poor, or fair	0
Good	10 (5)
Very good	34 (16)
Excellent	169 (79)
Any barriers to health care^b^	42 (20)
Any competing needs at baseline	27 (13)
Distance to clinic, kilometers	
< 5	108 (51)
5–10	64 (30)
> 10	40 (19)
Unknown	1 (0.5)
Years since initiation of ART at time of COVID-19 interview, *n* = 206	2 [1–4]
Chronic conditions - any one or more of: hypertension, diabetes, asthma	7 (3)
Sources of information on COVID-19^c^	
Radio	158 (74)
Television	156 (73)
Clinic materials/staff	46 (22)
Social media	28 (13)
At work	24 (11)
Friends/family	22 (10)
Newspaper/news website	17 (8)
Posters	11 (5)
DOH/Government website	6 (3)
Other	25 (12)
COVID-19 medical mistrust summary score, *n* = 144^d^	9 [6–13]
COVID-19 stigma summary score, *n* = 207	2 [0–5]
Recommended daily activities^e^ changed due to COVID-19	
None	43 (20)
1 activity	39 (18)
≥ 2 activities	131 (62)

*DOH* Department of Health

^a^According to the South Africa National Department of Education, ‘some high school’ refers to having started high school but not completing through Grade 12; tertiary level refers to any type of education pursued beyond the high school level. This includes diplomas, undergraduate and graduate certificates, and associate’s, bachelor’s, master’s and doctoral degrees

^b^Any one or more barriers to health care in the categories of service, financial, personal health, logistical, or structural. All other respondents reported no barriers to care

^c^Multiple answer choices allowed

^d^69 participants were missing data for one or more component questions of the medical mistrust summary score

^e^Activities falling within the recommendations of the South Africa National Department of Public Health including physical distancing (avoiding large gatherings, not hugging other people, avoiding people who present with symptoms, avoiding public transport, not going outside), wearing masks, hygiene (washing hands, not touching face, using hand sanitizer frequently)

Nearly three-quarters of participants reported radio (158, 74%) and television (156, 73%) as their main sources of information on COVID-19, followed by clinic materials or clinic staff (46, 22%) and social media (28, 13%) (Table 1). The median summary score for medical mistrust related to COVID-19 was 9 (IQR 6–13) on a scale from 0 to 28, with higher scores indicating greater medical mistrust. The median summary score for stigma related to COVID-19 was 2 (IQR 0–5) on a scale from 0 to 24, with higher scores indicating greater stigma. The majority of participants (170, 80%) changed at least one recommended daily activity because of COVID-19 (Table 1).

### Vaccine hesitancy

**Fig. 1:**
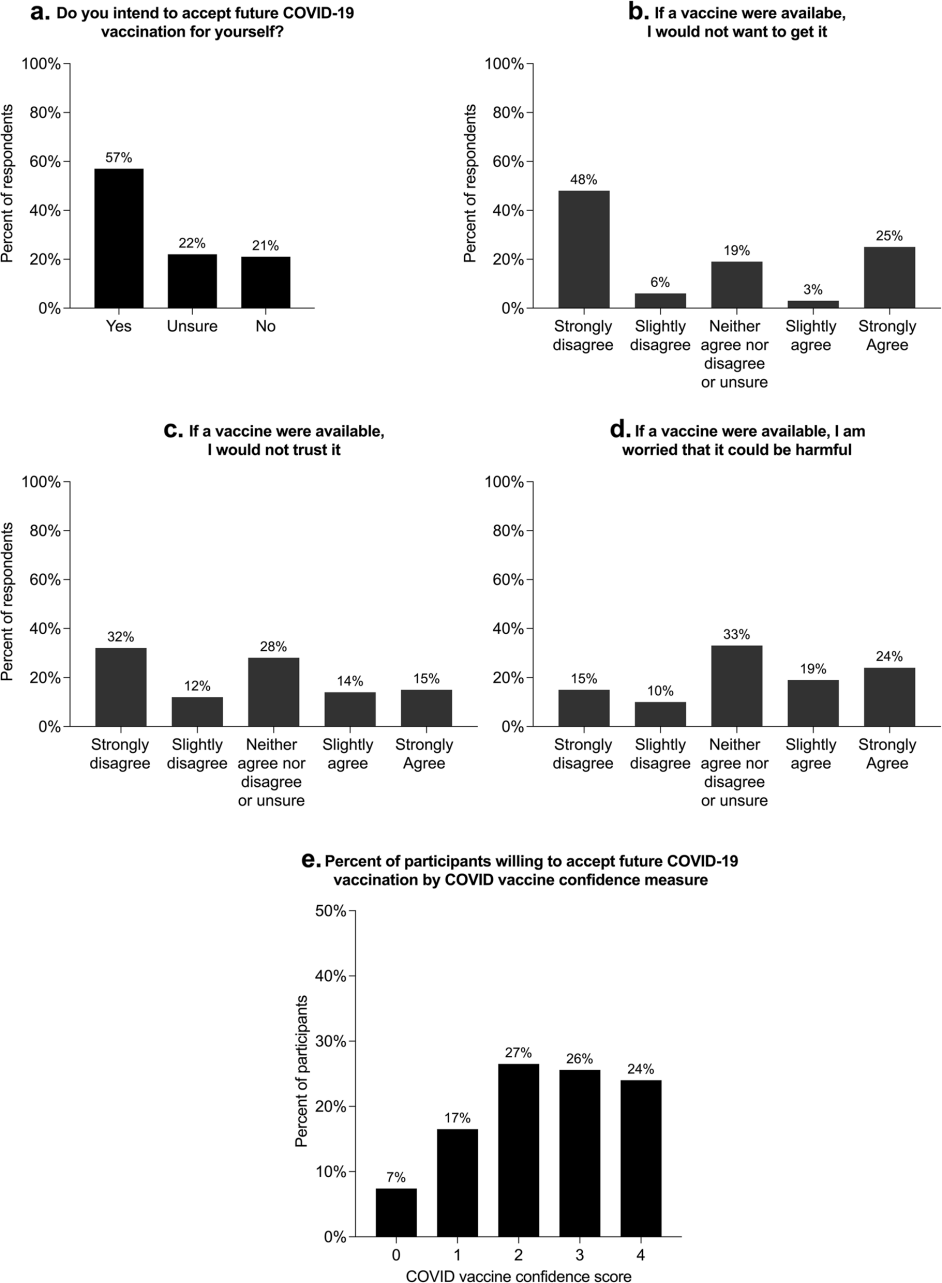
Distribution of responses to questions regarding COVID-19 vaccination. **e** Shows the percentage of participants who answered “Yes” to the question, “Do you intend to accept future COVID-19 vaccination for yourself?” by score on the COVID-19 vaccine confidence summary measure. The COVID-19 vaccine confidence summary measure was created by assigning one point each for those reporting no concerns regarding COVID-19 vaccination and disagreeing with each of the following statements: “if a vaccine were available to prevent COVID-19 in the future, I: ‘would not want to get it’, ‘would not trust it’, or ‘am worried that it could be harmful’”. The measure has a possible score range of 0–4, with higher scores indicating greater COVID-19 vaccine confidence. Cochran-Armitage test for trend *p* < 0.001

One hundred and seventy-eight (84%) participants were not vaccinated against influenza in the last influenza season, with 51 (29%) stating that they did not think the influenza vaccine was needed. More than half of all participants (121, 57%) responded that they are willing to accept future COVID-19 vaccination for themselves, with an additional 46 (22%) stating they were unsure if they would accept COVID-19 vaccination (Fig. 1). Similarly, 113 participants (53%) disagreed or strongly disagreed that if a vaccine were available to prevent COVID-19, they would not want to get it. Sixty participants (28%) agreed or strongly agreed that if a vaccine were available to prevent COVID-19, they would not trust it, while 90 participants (42%) agreed or strongly agreed that they would worry it could be harmful. The most common concerns regarding a COVID-19 vaccine were fear of side effects (*n* = 42, 20%), fear of getting associated COVID-19 illness from the vaccine (*n* = 32, 15%), and wanting to wait until the vaccine is tested by others (*n* = 25, 12%). Seventy-nine participants (37%) stated that they had no concerns regarding a future COVID-19 vaccine. Higher scores (greater confidence) on the summary COVID-19 vaccine confidence measure were associated with willingness to accept COVID-19 vaccination (Cochran-Armitage test for trend *p* < 0.001, Fig. 1e). Cronbach’s alpha for the summary COVID-19 vaccine confidence measure was 0.73, indicating good internal consistency.

Regarding general vaccine confidence, a majority of participants (203, 96%) strongly agreed that vaccines are important for children to have, 163 (77%) strongly agreed that overall they think vaccines are safe, 165 (79%) strongly agreed that vaccines are effective, and 180 (90%) strongly agreed that vaccines are compatible with their religious beliefs (Table 2).

**Table 2:** Vaccination concerns and vaccine confidence; *n* = 213 unless otherwise specified

	n, (%)
Concerns regarding future COVID-19 vaccine	
No concerns	79 (37)
Fear of side effects	42 (20)
Fear of getting associated COVID-19 illness	32 (15)
Want to wait until vaccine is tested by others	25 (12)
Worried about the origins of the vaccine	13 (6)
Cost of vaccine will be high	8 (4)
Expect long vaccination site wait time/queues	8 (4)
Do not think the vaccine will be effective	3 (1)
Vaccine is unnecessary because COVID-19 symptoms are mostly mild	1 (0.5)
Vaccine is unnecessary because biological (natural) immunity is better	1 (0.5)
Expect long distance to vaccination site	1 (0.5)
Other	9 (4)
Vaccinated against influenza in last influenza season	
Yes	32 (15)
No	178 (84)
Unsure	3 (1)
Refused	0
**Vaccine confidence measures**^a^	
Vaccines are important for children to have, *n* = 211	
Strongly disagree	1 (0.5)
Neither agree nor disagree or unsure	3 (1)
Slightly agree	4 (2)
Strongly agree	203 (96)
Overall, I think vaccines are safe, *n* = 211	
Strongly disagree	1 (0.5)
Disagree	1 (0.5)
Neither agree nor disagree or unsure	21 (10)
Slightly agree	25 (12)
Strongly agree	163 (77)
Overall, I think vaccines are effective, *n* = 210	
Strongly disagree	1 (0.5)
Disagree	1 (0.5)
Neither agree nor disagree or unsure	17 (8)
Slightly agree	26 (12)
Strongly agree	165 (79)
Vaccines are compatible with my religious beliefs, *n* = 200	
Strongly disagree	8 (4)
Neither agree nor disagree or unsure	6 (3)
Slightly agree	6 (3)
Strongly agree	180 (90)

^a^Adapted from Larson et al. 2016

In a multivariable analysis controlling for sex, older age was associated with willingness to accept COVID-19 vaccination (aOR 1.75 for every 10-year increase in age, 95% CI 1.10–2.78, *p* = 0.02). Reporting social media as a source of information on COVID-19 was associated with lower willingness to accept vaccination (aOR 0.30, 95% CI 0.11–0.84, *p* = 0.02), as was having a summary medical mistrust score at or above the median (aOR 0.21, 95% CI 0.093–0.45, *p* < 0.001) (Table 3). COVID-19 vaccination acceptance was not associated with sex, other sources of information on COVID-19, primary concerns about the COVID-19 pandemic, activities changed due to the COVID-19 pandemic, COVID-19 stigma, or recent vaccination against influenza (Table 3). COVID-19 vaccination acceptance was additionally not associated with travel distance, cost, time, or form of transport to clinic, change in motivation to take ART, household economic impact, changes made in household due to financial hardship, food insecurity, mental health, or perceived stress score (data not shown).

**Table 3:** Factors associated with intent to accept COVID-19 vaccination

	Intend to accept COVID-19 vaccination, n (%)		Unadjusted OR (95% CI)	***p***-value, univariate model	Adjusted OR (95% CI), ***n*** = 144	***p***-value, multivariable model
	No/Unsure	Yes				
Gender						
Female	68 (44)	85 (56)	0.80 (0.43–1.47)	0.47	0.89 (0.36–2.22)	0.81
Male	23 (39)	36 (61)	Ref	Ref	Ref	Ref
Age category						**0.018**
18–25	13 (68)	6 (32)	0.19 (0.063–0.58)	**0.007**	1.75 (1.10–2.78)	
26–40	59 (46)	69 (54)	0.48 (0.26–0.91)		per 10 year increase in age	
> 40	19 (29)	46 (71)	Ref			
Sources of information on COVID-19						
Radio	60 (38)	97 (62)	2.09 (1.12–3.89)	**0.021**	2.21 (0.94–5.21)	0.071
Television	71 (46)	84 (54)	0.64 (0.34–1.20)	0.16		
Social media	20 (71)	8 (29)	0.25 (0.11–0.60)	**0.002**	0.30 (0.11–0.84)	**0.022**
Friends/family	10 (45)	12 (55)	0.89 (0.37–2.17)	0.80		
Clinic materials/staff	17 (37)	29 (63)	1.37 (0.70–2.69)	0.36		
Median mistrust summary score, *n* = 144						
Below median	16 (24)	50 (76)	Ref	Ref	Ref	Ref
At or above median	47 (60)	31 (40)	0.21 (0.10–0.44)	**< 0.001**	0.21 (0.093–0.45)	**< 0.001**
Concerns about the COVID-19 pandemic						
Becoming infected myself	15 (42)	21 (58)	1.06 (0.51–2.20)	0.87		
Family member becoming infected	8 (57)	6 (43)	0.54 (0.18–1.62)	0.27		
Unable to work	28 (39)	44 (61)	1.29 (0.72–2.29)	0.40		
Food running out	6 (46)	7 (54)	0.87 (0.28–2.68)	0.81		
Money running out	13 (38)	21 (62)	1.26 (0.59–2.67)	0.55		
Death	9 (39)	14 (61)	1.19 (0.49–2.89)	0.70		
No concerns	18 (56)	14 (44)	0.53 (0.25–1.13)	0.10		
Recommended^a^ daily activities changed due to COVID-19 pandemic						
0	15 (36)	27 (64)	Ref	0.25		
1	21 (54)	18 (46)	0.48 (0.20–1.16)			
≥ 2	55 (42)	76 (58)	0.77 (0.37–1.58)			
COVID-19 Stigma summary score, *n* = 207						
Below median	36 (40)	54 (60)	Ref	Ref		
At or above median	55 (47)	62 (53)	0.75 (0.43–1.31)	0.31		
Vaccinated against influenza						
Yes	9 (28)	23 (72)	2.14 (0.94–4.88)	0.071		
No or unsure	82 (46)	98 (54)	Ref	Ref		

^a^Changes in activity falling within the recommendations of the South Africa National Department of Public Health

## Discussion

In a cohort of people living with well-controlled HIV in South Africa accessing ART through a decentralized medication distribution program, over half of individuals reported that they would be willing to accept a COVID-19 vaccine. Our sample of PLWH on ART is 72% female, which is similar to the 69% female national population of PLWH on ART in South Africa [27]. Increasing age was associated with higher odds of willingness to accept COVID-19 vaccination. The majority of participants received information about COVID-19 from television or radio; those who reported receiving most of their information about COVID-19 from social media were less likely to express willingness to accept COVID-19 vaccination than those who did not report social media as a source of information. Individuals with higher medical mistrust related to COVID-19 had lower odds of willingness to accept COVID-19 vaccination. While only a minority of participants were vaccinated against influenza in the last influenza season, overall vaccine confidence was high in this cohort.

While slightly more than half of participants in this cohort were willing to accept future COVID-19 vaccination for themselves, this rate is inadequate for the achievement of herd immunity in South Africa, which will require the vaccination of at least 70% of eligible adults. Other studies have reported a wide range of willingness to accept COVID-19 vaccination in sub-Saharan Africa, from 15% in Cameroon [28] to 56% in the Democratic Republic of the Congo [29]; 79% of 15,000 adults in 15 African countries stated they would accept a COVID-19 vaccine if it were deemed safe and effective [30]. Of note, while some studies have identified a relationship between a nation’s economic level and willingness to accept COVID-19 vaccination [31], in South Africa the greater concern is currently that low vaccination uptake may impede economic recovery due to recurrent COVID-19 surges, as the rate of full vaccination is at 48% as of March 2022, below the goal for the adult population [11].

Several studies among PLWH in high-income countries have found similar or lower rates of willingness to accept COVID-19 vaccination compared to our study. In a study conducted among Black American PLWH early in the COVID-19 pandemic, 32% stated that if a vaccine were available, they would not want to get it, and 54% endorsed any vaccine hesitancy belief, which was in turn associated with higher levels of medical mistrust [18]. In France, 71% of surveyed PLWH exhibited vaccine hesitancy [32]. Despite 43% of our cohort indicating they were unsure or were not willing to accept COVID-19 vaccination, the overall vaccine confidence, utilizing measures from a 67-country survey of vaccine confidence [17], was high in this cohort. Rates of agreement were over 70% for belief in vaccine safety and effectiveness, and at least 90% for belief that vaccines are important for children to have and that vaccines are compatible with participants’ religious beliefs. These results suggest that concerns specifically regarding COVID-19 vaccination, rather than a lack of general vaccine confidence, are primarily contributing to the rates of vaccine hesitancy identified in this study.

Fear of side effects from a COVID-19 vaccine was the most common concern regarding COVID-19 vaccination in this cohort, similar to previous studies of COVID-19 vaccine hesitancy and intent [28, 30, 32–34]. Previous concerns about vaccine safety have challenged the success and effectiveness of other vaccination programs in Africa, resulting in an increase in polio incidence in Nigeria [35]. Multinational clinical trials and post-vaccination monitoring surveys have revealed a low rate of serious side effects from the approved COVID-19 vaccines [36–38]. Strategic, evidence-based communication strategies regarding vaccine safety will need to be a priority for improving vaccine acceptance [39, 40].

Results of this study provide additional insight into determinants of willingness to accept COVID-19 vaccination among PLWH. We found that PLWH who are older are more likely to accept vaccination, consistent with previous findings regarding COVID-19 vaccine intent among people with and without HIV [41]. This finding may suggest that those who perceive themselves to be at higher risk of severe illness with COVID-19 are more likely to accept vaccination, even among this population with HIV who may already be at increased risk for complications. Further, higher medical mistrust related to COVID-19 was associated with lower willingness to be vaccinated. Medical mistrust, which can have roots in historical mistreatment by the healthcare system and in systemic racism, is also fueled by misinformation [25], which has been widespread since the beginning of the COVID-19 pandemic [42, 43]. Medical mistrust and conspiracy theories, a related concept, have also been associated with lower willingness to accept COVID-19 vaccination in other studies among people with and without HIV [18, 29, 34, 44]. The second most common concern about the COVID-19 vaccine in our study was fear of getting COVID-19 from the vaccine itself, reflecting a common conspiracy belief. Notably, social media as a main source of information on COVID-19, which we found to be negatively associated with willingness to accept COVID-19 vaccination, has also been associated with COVID-19 misinformation/mistrust [42, 45] and with decreased willingness to accept COVID-19 vaccination in other studies [44].

More than 80 % of participants were not vaccinated against influenza in the last influenza season, despite a recommendation from the South African Department of Health for annual influenza vaccination for PLWH [46]. In light of overall high vaccine confidence, this result suggests either inadequate public health messaging and education regarding the influenza vaccine or a lack of access to the influenza vaccine among this population. In light of competing health priorities, countries such as South Africa may have limited supply of influenza vaccines [47], or infrastructure for vaccine distribution to the adult population may be insufficient. The rapid distribution of COVID-19 vaccination may therefore need to be accompanied by the development of infrastructure and by widespread public health messaging assuring access to and availability of the vaccine.

This study is among the first to evaluate willingness to accept COVID-19 vaccine among PLWH in South Africa, which has the world’s largest population of PLWH [48]. Understanding this population’s perspective is important to inform vaccination efforts as the South African Department of Health has begun rolling out COVID-19 vaccines. Only PLWH already successfully engaged in medical care were included, potentially decreasing the generalizability of our results to the general population of PLWH. However, this is an important population to assess, especially in South Africa considering the high HIV prevalence and the potential risks of not reaching effective vaccination rates in this population. Further, our questions assessing stigma and medical mistrust may not accurately assess all dimensions of these constructs for COVID-19, and were developed before any tools had been validated for this purpose. However, as we did find an association between medical mistrust and willingness to accept COVID-19 vaccination, we believe that we captured at least some aspects of these constructs accurately. Additionally, the study was conducted between the first and second waves of the pandemic in South Africa and before the introduction of COVID-19 vaccination. Due to limitations in research resources during the pandemic, the interviews took place over the course of several months, making it possible that participant responses changed over time, both with regards to stigma and medical mistrust and willingness to accept vaccination. Given the dynamic nature of the COVID-19 pandemic and the rapid roll-out of vaccination in many parts of the world, re-evaluating vaccine intention now that vaccination is underway in South Africa might bring a new dimension to the current findings. Nonetheless, the study has demonstrated that over half of PLWH engaged in care are willing to accept a future COVID-19 vaccine, providing a baseline against which to compare changes over time as vaccination is introduced in South Africa, and an understanding of areas of concern that would benefit from targeted communication and education efforts to increase COVID-19 vaccine uptake in this population.

## Conclusions

Among people living with HIV enrolled in a decentralized ART delivery program in South Africa, over half of individuals reported that they are willing to accept a COVID-19 vaccine. Fear of side effects from a COVID-19 vaccine was the most common concern regarding COVID-19 vaccination. Higher age was associated with willingness to accept COVID-19 vaccination, while higher medical mistrust related to COVID-19 and obtaining information on COVID-19 from social media were associated with lower odds of willingness to accept COVID-19 vaccination. While nearly half of participants were either unsure or not willing to accept COVID-19 vaccination, overall vaccine confidence was high. Our results suggest that communication strategies reassuring safety and efficacy of COVID-19 vaccines and addressing sources of misinformation on COVID-19 will be important to support COVID-19 vaccine uptake in this population.

## Supplementary Information

**Additional file 1.** COVID-19 telephone questionnaire.

**Additional file 2.** Baseline questionnaire for parent study.

## Data Availability

Data are not posted online due to potentially identifiable information but are available from the principal investigator upon request.

## Abbreviations

PLWH: People living with HIV
ART: Antiretroviral therapy
WHO: The World Health Organization
SAGE: Strategic Advisory Group of Experts
CCMDD: Central Chronic Medicine Dispending and Distribution
KZN: KwaZulu-Natal
