# Supplementary material for: Willingness to accept COVID-19 vaccination among people living with HIV in a high HIV prevalence community
Source: BMC Public Health. 2022 Jun 22;22:1239. doi: 10.1186/s12889-022-13623-w (PMC9214683; doi:10.1186/s12889-022-13623-w)
Supplement: Supplementary file 1 — Additional file 1. COVID-19 telephone questionnaire. [file 12889_2022_13623_MOESM1_ESM.pdf]

Today's Date: day / month / year   Study ID: ____________

Interviewer ID: ____________   Location/Facility: ____________

South Africa's new Central Chronic Medicine Dispensing and Distribution Program: assessing the clinic, patient and community impact

Participant Phone Interview
CCMDD COVID-19 Supplement

**A. PATIENT DATA (Collected from REDCAP)**			
A1	Participant name:	[Pulled from current form]	
A2	Participant SA ID number:	__ __ __ __ __ __ __ __ __ __ __ __ __<br>*Missing: 9s throughout* [Pulled from current form]	
A3	Participant Date of Birth:	DD / MM / YYYY [Pulled from current form]	
A4	Participant Cell number:	(__ __ __) __ __ __ __ __ __ __<br>*Missing: 9s throughout* [Pulled from current form]	
A5	Did the participant provide COVID supplement interview?	Yes……………………………………………………1<br>No……………………………………………………...2	 →END

**B. PREPOPULATION: CCMDD DATA (Collected from REDCAP)**			
B1	Date enrolled into CCMDD program:	[Pulled from current form]	
B2	Name of chosen pick-up point:	[Pulled from current form]	

C. BEGINNING OF PHONE INTERVIEW

RA: Hello, this is <u>RA's Name</u> calling from Ithembalabantu Clinic. Is this Mr/Miss/Mrs John Doe?

Patient: Yes, this is John Doe.

RA: Sir/ Ma'am, thank you. To protect the safety and privacy of our patients, I would just like to confirm your date of birth. Can you please tell me your date of birth?

If the patient is unable to confirm the correct date of birth, the RA will thank the patient for their time, and indicate that the date the patient has provided does not match the date on the study records and, to avoid any breach of confidentiality, cannot proceed with the interview.

Today's Date:	day / month / year	Study ID:	____________
Interviewer ID:	____________	Location/Facility:	____________

If the patient is able to provide the correct date of birth, the RA will thank the patient and proceed with the phone-call.

RA: The purpose of this survey is to better understand the experience of CCMDD participants during the time of the COVID-19/coronavirus outbreak.

All information that you share with me today is completely confidential and will only be used to help us with our research. Once we complete our study, we will destroy the link between your name and your answers to our interview questions. We will not release any information that will allow linking of your name to your specific comments or to your specific clinic.

The interview is completely voluntary, you can stop participating at any time, and you can skip any questions you'd rather not answer.

Finally, this interview will take about 20-30 minutes. At the end, we will give you 50 South African Rand for participating. Do you voluntarily choose to enroll with the understanding that you may decline or stop answering this questionnaire at any time?

C1	Did the participant confirm DOB?	0, No<br>1, Yes	→ END
C2	Participant voluntarily chose to enroll after being read the informed consent script?	No……………………………………………….0<br>Yes………………………………………………1	→ END
**D. PHONE: CCMDD**			
We are first going to ask you about your experiences with getting medication through the CCMDD program.			
D1	Are you currently getting ARV medication through the CCMDD program?	No……………………………0<br>Yes…………………………...1<br>Refused…………….……….-1	→D6<br><br>→D6
D2	How far is your home from your usual CCMDD pick-up point?	Less than 5 kilometers……....1<br>5 to 10 kilometers…………...2<br>10 to 30 kilometers………….3<br>More than 30 kilometers……4<br>Refused………………….... -1<br>Unknown……………...……-2	

Today's Date: day / month / year Study ID: ____________________

Interviewer ID: ____________________ Location/Facility: ____________________

D3	How do you usually travel to your CCMDD pick-up point?	Walk……………………………………..1<br>Public transport (bus, taxi)…………………..2<br>Private transport……………………………..3<br>Other ………….…………………………..99<br>If OTHER, specify:______________<br>Refused…………………………………… -1	
D4	How long does it usually take for you to get to your CCMDD pick-up point and back (round trip)?	Time available……………………………...1<br>__ __ hours __ __ minutes<br>Refused…………………………………… -1<br>Unknown…………………………………..-2	
D5	How much money in total does it usually cost you to get to your CCMDD pick-up point and back (round trip)?	__ __ __ South African Rand<br>Refused……………………………………-1	
D6	When was the last time you picked up your ARV medication?	_ _ / _ _ _ / _ _ _ _<br>(dd / mmm / yyyy)<br>[Day: 01-31; *Unknown if unknown* (pull down)<br>Month: Jan, Feb, Mar, Apr, May, Jun, Jul, Aug, Sep, Oct, Nov, Dec; *Unknown if unknown* (pull down)<br>Year: 2015-2023; *Unknown if unknown* (pull down)]<br>*Date check – date should not be after today*	
D7	Where did you last pick up your ARV medication?<br>*Pull down*	D Clinic……………………………………………1<br>G Clinic……………………………………………2<br>U21 Clinic…………………………………………3<br>K Clinic……………………………………………4<br>Gateway Clinic…………………………………….5<br>Q Clinic……………………………………………6<br>L Clinic……………………………………………7	

Today's Date: day / month / year Study ID: ___________________

Interviewer ID: ___________________ Location/Facility: ___________________

		H Clinic……………………………………………8<br>N Clinic……………………………………………9<br>AA Clinic…………………………………………..10<br>Section D Bahle (NGO support group)……………11<br>St Phillips Anglican Church Umlazi J Section……12<br>P Section Gospel church…………………………..13<br>Z taxi rank…………………………………………14<br>AA Counsellors office……………………………..15<br>BB Siyaphaphila centre……………………………16<br>CC creche…………………………………………..17<br>Dr Sithole's rooms (Mega City)……………………18<br>Nyanda……………………………………………..19<br>MobiMed…………………………………………...20<br>Megacity Clicks……………………………………21<br>Megacity Spar……………………………………...22<br>Clicks Chatsworth………………………………….23<br>Clicks Galleria……………………………………..24<br>Clicks Westguard…………………………………..25<br>Clicks KwaMnyandu Shopping Center……………26<br>Clicks Workshop…………………………………...27<br>Ithembalabantu Clinic……………………………...28<br>Pele Box……………………………………………29<br>Other………………………………………………..99<br>If OTHER, specify: ________<br>Refused…………………………………………….-1<br>Unknown…………………………………………...-2	
D8	Since the last time you picked up your ARV medication, have you run out of pills?	No……………………………0<br>Yes……………………………1<br>Refused……………………….-1	
D9	Did you miss or delay your last scheduled ARV medication pick-up?	0, No<br>1, Yes – delayed pick-up<br>2, Yes – missed pick-up	→ D11

Today's Date: day / month / year Study ID: ___________________

Interviewer ID: ___________________ Location/Facility: ___________________

		-1, Refused	→ D11
D10	If you have missed picking up your ARV medication, what are the reasons?<br>*(Check all that apply)*	Transportation availability……………………………1<br>Transportation cost…………………………………2<br>Safety concerns……………………………….…….3<br>COVID-19 infection risk…………………………...4<br>Busy with work…………………………………..5<br>Busy with family……………………………………6<br>Illness – self…………………………………………7<br>Illness – family…………………………………...8<br>Concern that pick-up point is closed………………..9<br>Pick-up point closed/no staff present………………10<br>Other………………………………………………...99<br>If OTHER, specify: ________<br>Refused……………………………………………..-1	
D11	Many people find it difficult to take all their HIV medications as prescribed. How many doses of your HIV medication have you missed in the last 7 days?	__ __ doses<br>Don't know……………………………………-2<br>Refused………………………………………..-1<br>[Drop down with numbers 00-14, -2, -1]	

Today's Date: day / month / year Study ID: ____________

Interviewer ID: ____________ Location/Facility: ____________

D12	When is your next scheduled date to pick up your ARV medication?	_ _ / _ _ _ / _ _ _ _<br>(dd / mmm / yyyy)<br>[Day: 01-31; *Unknown if unknown* (pull down)<br>Month: Jan, Feb, Mar, Apr, May, Jun, Jul, Aug, Sep, Oct, Nov, Dec; *Unknown if unknown* (pull down)<br>Year: 2015-2023; *Unknown if unknown* (pull down)]<br>*Date check – date should not be before today*	
D13	Moving forward, do you have any new concerns about picking up your ARV medication?<br>*(Check all that apply)*	Transportation availability…………………………1<br>Transportation cost…………………………………2<br>Safety concerns…………………………….…….3<br>COVID-19 infection risk…………………………...4<br>Busy with work……………………………………..5<br>Busy with family……………………………………6<br>Illness – self………………………………………7<br>Illness – family……………………………………...8<br>Concern that pick-up point is closed………………..9<br>Pick-up point closed/no staff present……………….10<br>Other………………………………………………...11<br>If OTHER, specify: ________<br>Refused……………………………………………..-1	
**E. PHONE: COVID-19 Knowledge and Stigma**			
We are now going to ask you about your current knowledge about the "novel coronavirus", the new virus called SARS-CoV-2, which causes a disease called "COVID-19", that has been impacting our community recently. For the rest of the questionnaire I will refer to this as "coronavirus".			
E1	Have you heard of coronavirus?	No…………………………….…0<br>Yes……………………………1<br>Refused……………………….-1	→F1<br><br>→F1

Today's Date:	day / month / year	Study ID:	___________________
Interviewer ID:	___________________	Location/Facility:	___________________

E2	Where do you get most of your information on coronavirus?<br>*(Check all that apply)*	Newspaper/news websites……1<br>Radio…………………………2<br>TV……………………………3<br>DOH/Government Website…...4<br>Social media…………………..5<br>Other websites………………...6<br>Friends/Family..……………...7<br>Posters………… ……….…...8<br>Clinics (materials, staff)….…...9<br>Schools…………..……………10<br>Church………………………...11<br>Billboards……………………..12<br>Traditional healers…………….13<br>Other…………………………..99<br>If OTHER, specify: ________<br>Refused………………………..-1	
E3	What symptoms do you associate with coronavirus?<br>*(Check all that apply)*	Cough………………………………….…1<br>Chest tightness/difficulty breathing…………...2<br>Sore throat…………………………………3<br>Muscle aches………………………………4<br>Fever………………………………………..5<br>Headache……………………………………6<br>Stomach pain………………………………..7<br>Congestion (runny nose)………………………8<br>Rash……………………………………………9<br>Joint aches…………………...10<br>Other…………………………99<br>If OTHER, specify: ________	

Today's Date: day / month / year Study ID: ____________________

Interviewer ID: ____________________ Location/Facility: ____________________

		Refused……………………………………….-1	
E4	If you were to have symptoms of coronavirus, what would you do?<br>*(Check all that apply)*	Stay at home…………………1<br>Call/go to clinic……………..2<br>Go to church…………………3<br>Go to traditional healer………4<br>COVID hotline………………5<br>DOH COVID WhatsApp……6<br>Other…………………………99<br>If OTHER, specify: ________<br>Refused……………………….-1	
**Vaccine Hesitancy**			
E5	Did you vaccinate yourself against seasonal influenza in 2019?	No……………………………0<br>Yes…………………………...1<br>Unsure..……………...………-2<br>Refused…………...………-1	 →E7<br>→E7<br>→E7
E6	If no, why not?<br>(instructions: leave open-ended, do not prompt or provide answer options)	Did not think it was needed………………………..……. …………1<br>Did not know where to get vaccination…………….......................2<br>Did not know where to get good/reliable information…………......3<br>Heard or read negative media……………………….....................4<br>Did not think the vaccine was effective…………………………...5<br>Did not think the vaccine was safe/concerned about side effects…6<br>Had a bad experience with previous vaccinator/health clinic……..7<br>Had a bad experience or reaction with previous vaccination……..8<br>Someone else told me they/their child had a bad reaction..………9<br>Fear of needles………...10<br>Not possible to leave other work (at home or other)………………11<br>Religious reasons…………………………………………………12<br>Other beliefs/traditional medicine………………………………..13	

Today's Date: day / month / year   Study ID: ____________

Interviewer ID: ____________   Location/Facility: ____________

		My doctor told me I am not a candidate for vaccination………..14<br>No time to get vaccinated…………………………………………15<br>I am allergic…………………………………………………..…16<br>Cost of vaccine………………………………………………17<br>Cost of travel to vaccination site………………….......................18<br>Distance to vaccination site………………………………………19<br>Vaccination site hours………..20<br>Vaccination site wait time……………………………….…21<br>Other……………………………………………………………..99<br>If OTHER, specify: ________<br>Refused………………………………………………………...-1	
E7	Do you intend to accept future COVID-19 vaccination for yourself?	No……………………………0<br>Yes…………………………...1<br>Unsure..…………….....………-2<br>Refused…………….....………-1	
E8	What are your concerns regarding a future COVID-19 vaccine?<br>(instructions: leave open-ended, do not prompt or provide answer options)	Fear of side effects……………………………….......................1<br>Fear of getting associated COVID-19 illness……….…………....2<br>Want to wait until vaccine is tested by others………....................3<br>Fear of taking vaccine while pregnant…..…………………………...4<br>Do not think the vaccine will be effective…………………………5<br>Vaccine is unnecessary because COVID-19 symptoms are mostly mild……………………………………………..……………6<br>Vaccine is unnecessary because biological (natural) immunity is better…………………………………………………………7<br>Cost of vaccine will be high…………………………………..…..8<br>Will not be able to afford cost of travel to vaccination site.………9<br>Expect long distance to vaccination site..10<br>Expect inconvenient vaccination site hours………………………11	

Today's Date: day / month / year   Study ID: ________

Interviewer ID: ________   Location/Facility: ________

		Expect long vaccination site wait time/queues..…………………12<br>Worried about the origins of the vaccine……….……. …………13<br>No concerns……………………………………………………98<br>Other…………………………………………………………..99<br>If OTHER, specify: ________<br>Refused…………………………………………………….....-1

Questions E9-E12 ask about any vaccine or vaccines in general. Questions E13-E15 are specific to the COVID-19 vaccine. Please tell us how much you agree or disagree with the following statements:

		Strongly disagree	Slightly disagree	Neither disagree nor agree or unsure	Slightly agree	Strongly agree	N/A
E9	Vaccines are important for children to have.	1	2	3	4	5	-99
E10	Overall, I think vaccines are safe.	1	2	3	4	5	-99
E11	Overall, I think vaccines are effective.	1	2	3	4	5	-99
E12	Vaccines are compatible with my religious beliefs	1	2	3	4	5	-99
E13	If a vaccine were available to prevent COVID-19, I would not want to get it.	1	2	3	4	5	-99
E14	If a vaccine were available to prevent COVID-19, I would not trust it.	1	2	3	4	5	-99
E15	If a vaccine were available to prevent COVID-19, I am	1	2	3	4	5	-99

Today's Date: day / month / year Study ID: ___________________

Interviewer ID: ___________________ Location/Facility: ___________________

	worried that it could be harmful.						
**COVID-19 Mistrust/Misinformation Scale**							
Please tell us how much you agree or disagree with the following statements:							
		Strongly disagree	Disagree	Not sure	Agree	Strongly agree	Prefer not to answer
E16	The government cannot be trusted to tell the truth about coronavirus.	1	2	3	4	5	-1
E17	I trust the public health measures that the government is taking to combat coronavirus.	1	2	3	4	5	-1
E18	Information about coronavirus is being withheld from the public.	1	2	3	4	5	-1
E19	I trust the information that I am hearing on the radio and TV about coronavirus.	1	2	3	4	5	-1
E20	I trust the information that I am reading about coronavirus.	1	2	3	4	5	-1
E21	I trust that the doctors and nurses in clinics know how to treat coronavirus.	1	2	3	4	5	-1
E22	I trust traditional healers more than clinic workers to treat coronavirus.	1	2	3	4	5	-1

Today's Date: day / month / year   Study ID: ____________________

Interviewer ID: ____________________   Location/Facility: ____________________

E23	I trust church leaders more than clinic workers to treat coronavirus.	1	2	3	4	5	-1

E24	What are your concerns about coronavirus?<br>*(Check all that apply)*	Becoming infected myself………………………..1<br>Family member becoming infected………………2<br>Lack of transport to work…………………………3<br>Lack of transport to clinic………………………...4<br>Lack of transport to CCMDD pick-up point……...5<br>Lack of transport elsewhere………………………6<br>Unable to work……………………………………7<br>Food running out…………………………………8<br>Money running out……………………………...9<br>Crime……………………………………………...10<br>Domestic violence………………………………11<br>Lack of information………………………………12<br>Stigma……………………………………………..13<br>Afraid to move……………………………………14<br>Unavailability of sanitizer/protective equipment….15<br>Other……………………………………………….99<br>If OTHER, specify: ________<br>Refused…………………………………………….-1	
E25	Have you changed any of your normal daily activities since learning about coronavirus?	No………………………….…0<br>Yes…………………………….1<br>Refused……………………….-1	→E16<br><br>→E16
E26	How have you changed your normal daily activities since learning about coronavirus?	Stopped taking taxis/public transport…...………1<br>Avoiding people who present symptoms……….2<br>Not greeting other people……………………….3	

Today's Date: day / month / year Study ID: ____________________

Interviewer ID: ____________________ Location/Facility: ____________________

	(Check all that apply)	Not hugging other people……………………….4<br>Visiting the clinic less…………………………...5<br>Visiting the pharmacy less………………………6<br>Not going to work………………………………7<br>Not going outside………………………………..8<br>Avoiding large gatherings………………………9<br>Avoiding family members………………………10<br>Avoiding friends…………………………………11<br>Self-isolation……………………………………..12<br>Washing hands more……………………………..13<br>Not touching face………………………………...14<br>Using hand sanitizer frequently…………………...15<br>Wearing a mask…………………………………...16<br>Living elsewhere…………………………………17<br>Other……………………………………………...99<br>If OTHER, specify: ________<br>Refused……………………………………………-1	
E27	Have you delayed picking up your ARV medication because of coronavirus?	No…………………………………………………0<br>Yes...……………………………………………1<br>Refused……………………………………..…...-1	→ E18<br><br>→ E18

Today's Date:	day / month / year	Study ID:	___________________
Interviewer ID:	___________________	Location/Facility:	___________________

E28	If you have delayed picking up your ARV medication, what are the reasons?<br>*(Check all that apply)*	Transportation availability…………………………1<br>Transportation cost…………………………………2<br>Safety concerns…………………………….……3<br>COVID-19 infection risk…………………………..4<br>Busy with work……………………………………..5<br>Busy with family……………………………………6<br>Illness – self…………………………………………7<br>Illness – family……………………………………...8<br>Concern that pick-up point is closed………………..9<br>Pick-up point closed/no staff present……………….10<br>Other…………………………………………………...99<br>If OTHER, specify: ________<br>Refused……………………………………………..-1	
E29	Has your experience picking up ARV medication changed in any way because of coronavirus?	No…………………………..…0<br>Yes……………………………1<br>Have not been to pick-up point recently….2<br>Refused……………………….-1	→E27<br><br>→E28<br>→E27
E30	Has your pick-up point location changed because of coronavirus?	No…………………………..…0<br>Yes……………………………1<br>Refused……………………….-1	→E21<br><br>→E21
E31	Where is your new pick-up point location?<br>*Drop down*	D Clinic……………………………………………1<br>G Clinic……………………………………………2<br>U21 Clinic…………………………………………3<br>K Clinic……………………………………………4<br>Gateway Clinic…………………………………….5<br>Q Clinic……………………………………………6<br>L Clinic……………………………………………7<br>H Clinic……………………………………………8<br>N Clinic……………………………………………9<br>AA Clinic…………………………………………….10	

Today's Date: day / month / year Study ID: ____________

Interviewer ID: ____________ Location/Facility: ____________

		Section D Bahle (NGO support group)……………11<br>St Phillips Anglican Church Umlazi J Section……12<br>P Section Gospel church…………………………..13<br>Z taxi rank………………………………………14<br>AA Counsellors office……………………………..15<br>BB Siyaphaphila centre……………………………16<br>CC creche…………………………………………17<br>Dr Sithole's rooms (Mega City)……………………18<br>Nyanda……………………………………………..19<br>MobiMed…………………………………………...20<br>Megacity Clicks……………………………………21<br>Megacity Spar……………………………………...22<br>Clicks Chatsworth………………………………….23<br>Clicks Galleria……………………………………..24<br>Clicks Westguard…………………………………..25<br>Clicks KwaMnyandu Shopping Center……………26<br>Clicks Workshop…………………………………...27<br>Ithembalabantu Clinic……………………………...28<br>Other………………………………………………..…99<br>If OTHER, specify: ________<br>Unknown…………………………………………-2<br>Refused……………………………………………-1	
E32	Has the amount of time it takes to get to your pick-up point changed because of coronavirus?	No change…………………………….…1<br>Yes, increased……………………………2<br>Yes, decreased……………………………3<br>Refused……………………….-1	
E33	Has the cost of getting to your pick-up point changed because of coronavirus?	No change …………………………….…1<br>Yes, increased……………………………2<br>Yes, decreased……………………………3<br>Refused……………………….-1	
E34	Has the wait time when picking up your ARV	No change…………………………….…1<br>Yes, increased……………………………2	

Today's Date:	day / month / year	Study ID:	___________________
Interviewer ID:	___________________	Location/Facility:	___________________

	medication changed because of coronavirus?	Yes, decreased……………………………3<br>Refused……………………….-1	
E35	How crowded is your ARV pick-up point now compared to before coronavirus?	About the same…………………………..1<br>More crowded……………………………2<br>Less crowded……………………………3<br>Refused……………………….-1	
E36	How were you treated by the pick-up point staff while picking up your ARV medications?	Worse than usual…………………………1<br>Same as usual……………………………..2<br>Better than usual…………………………..3<br>Refused…………………………………….-1	
E37	Did your pick-up point have your ARV medication in stock?	No…………………………..0<br>Yes……………………………1<br>Refused……………………….-1	
E38	What screening for COVID did you experience at your pick-up point? *(Check all that apply)*	1, No screening<br>2, Symptom screening<br>3, Temperature screening<br>4, Nasal swab<br>5, Other<br>If OTHER, please list: ______________________<br>-1, Refused	
E39	Do you think the date of your next ARV medication pick-up will change because of coronavirus?	No…………………………..0<br>Yes……………………………1<br>Unsure……………………...-2<br>Refused……………………….-1	
E40	Has the coronavirus or associated lockdown regulations affected your	1, No change<br>2, Yes, increased motivation to take ARV medication regularly	→ E31

Today's Date: day / month / year Study ID: ____________________

Interviewer ID: ____________________ Location/Facility: ____________________

	motivation to take your ARV medication regularly?	3, Yes, decreased motivation to take ARV medication regularly<br>-1, Refused	→ E31
E41	If YES, why?	1, Difficulty with picking up ARV medication<br>2, Fear of getting very sick from COVID-19<br>3, Fear of taking pills<br>4, Concern about my overall health during the pandemic<br>5, Feeling hopeless<br>6, Fear of going into clinic to collect medication<br>99, Other<br>If OTHER, please describe: ____________________<br>-1, Refused	
E42	Do you think some people are more at-risk of getting coronavirus?	No…………………………..0<br>Yes……………………………1<br>Unsure………………………...-2<br>Refused……………………….-1	→ E33<br><br>→ E33<br>→ E33

Today's Date: day / month / year Study ID: ____________

Interviewer ID: ____________ Location/Facility: ____________

E43	Who do you think is more at-risk of getting coronavirus? *(Check all that apply)*	Elderly………………………………………………1<br>Immunocompromised………………………………2<br>People living with HIV……………………………...3<br>Pregnant women……………………………………4<br>People living with TB………………………………5<br>Taxi drivers…………………………………………6<br>Healthcare workers…………………………………7<br>Grocery store workers………………………………8<br>Sex workers…………………………………………9<br>Children……………………………………………10<br>People who smoke…………………………………11<br>People who have travelled…………………………12<br>People with cancer…………………………………13<br>People with diabetes………………………………..14<br>Other………………………………………………..99<br>If OTHER, specify: ________<br>Refused……………………………………………..-1	
E44	Do you think some people are more at-risk of getting very sick from coronavirus?	No…………………………………………………..0<br>Yes…………………………………………………1<br>Unsure………………………………………………-2<br>Refused……………………………………………..-1	→ E35<br><br>→ E35<br>→ E35

Today's Date: day / month / year Study ID: ____________

Interviewer ID: ____________ Location/Facility: ____________

E45	Who do you think is more at-risk of getting very sick after having coronavirus?<br>*(Check all that apply)*	Elderly……………………………………………1<br>Immunocompromised………………………………2<br>People living with HIV…………………………...3<br>Pregnant women……………………………………4<br>People living with TB………………………………5<br>Taxi drivers…………………………………………6<br>Healthcare workers…………………………………7<br>Grocery store workers………………………………8<br>Sex workers…………………………………………9<br>Children……………………………………………10<br>People who smoke…………………………………11<br>People who have travelled…………………………12<br>People with cancer…………………………………13<br>People with diabetes………………………………..14<br>Other………………………………………………..99<br>If OTHER, specify: ________<br>Refused……………………………………………..-1					
**COVID-Related Stigma**							
Please tell us how much you agree or disagree with the following statements:							
		Strongly disagree	Disagree	Not sure	Agree	Strongly Agree	Prefer Not to Answer
E46	If someone I knew had coronavirus and recovered, I would be afraid to visit them.	1	2	3	4	5	-1
E47	If I had coronavirus, it would be difficult to tell other people.	1	2	3	4	5	-1

Today's Date:	day / month / year	Study ID:	___________________
Interviewer ID:	___________________	Location/Facility:	___________________

E48	If I had coronavirus, I would feel ashamed.	1	2	3	4	5	-1
E49	If I had coronavirus, my friends and family will be angry with me.	1	2	3	4	5	-1
E50	A person with coronavirus must have done something wrong and deserves to be punished.	1	2	3	4	5	-1
E51	A person with coronavirus is cursed.	1	2	3	4	5	-1
**F. PHONE: Recent Experiences Seeking Care**							
We are now going to ask you about any recent experiences you have had with seeking care.							
F1	Have you tried to seek care in the past month for any reason other than HIV?	No………………………….…0<br>Yes……………………………1<br>Refused……………………….-1					→ G1<br><br>→ G1
F2	What did you seek care for during your most recent clinic visit?	TB……………………..…………1<br>Chronic care (such as diabetes, high blood pressure)…..……………..………2<br>Sexual and reproductive health (such as contraceptive care, antenatal care, STI symptoms...3<br>Cold/flu……………………………..4<br>Other………………………….....99<br>If OTHER, specify: ________<br>Refused………………………….-1					
F3	Where did you go for your most recent clinic visit?	AA Clinic………………………………………1<br>D Clinic……………………………………..2<br>G Clinic……………………………………..3<br>H Clinic……………………………………...4					

Today's Date: day / month / year   Study ID: ___________________

Interviewer ID: ___________________   Location/Facility: ___________________

		K Clinic……………………………………………5<br>L Clinic……………………………………………6<br>N Clinic……………………………………………7<br>Q Clinic……………………………………………8<br>U21 Clinic…………………………………………9<br>Gateway Clinic……………………………………10<br>Ithembalabantu Clinic…………………………..11<br>Other………………………………………………99<br>If OTHER, specify: ________<br>Refused……………………………………………-1	
F4	How far did you travel to attend your most recent clinic visit?	Less than 5 kilometers……....1<br>5 to 10 kilometers…………...2<br>10 to 30 kilometers…………3<br>More than 30 kilometers……4<br>Refused…………………... -1<br>Unknown…………..……-2	
F5	What mode of transport did you use to attend your most recent clinic visit?	Walked………………………………………..1<br>Public transport (bus, taxi)…………………..2<br>Private transport……………………………..3<br>Other ………….……………………………..99<br>If OTHER, specify:______________<br>Refused…………………………………… -1	
F6	How much time did it take for you to get to your most recent clinic visit?	Time available…………………………...1<br>Refused…………………………………… -1<br>Unknown………………………………..-2<br>__ __ hours __ __ minutes	

Today's Date: day / month / year Study ID: ____________________

Interviewer ID: ____________________ Location/Facility: ____________________

F7	How did the wait length during your most recent clinic experience compare to previous visits?	Similar wait length……………………………1<br>Longer wait……………………………………2<br>Shorter wait……………………………………3<br>Refused…………………………………………..-1	
F8	How crowded was the clinic during your most recent clinic experience compared to previous visits?	Same…………………………………………..1<br>More crowded…………………………………2<br>Less crowded………………………………….3<br>Refused…………………………………………..-1	
F9	How were you treated by clinic staff during your most recent clinic experience compared to previous visits?	Worse than usual……………………………..1<br>Same as usual………………………………...2<br>Better than usual……………………………...3<br>Refused………………………………………-1	
F10	What screening for COVID did you experience during your most recent clinic experience? *(Check all that apply)*	1, No screening<br>2, Symptom screening<br>3, Temperature screening<br>4, Nasal swab<br>5, Other<br>If OTHER, please list: ______________________<br>-1, Refused	
**G. PHONE: Competing Needs Questions**			
G1	Over the past four weeks, have coronavirus and associated regulations affected the ability of you or your household to earn money?	0, No<br>1, Yes<br>-2, Unsure<br>-1, Refused	→ G3<br><br>→ G3<br>→ G3

Today's Date: day / month / year Study ID: ____________________

Interviewer ID: ____________________ Location/Facility: ____________________

G2	If YES, how?<br>*(Check all that apply)*	1, Additional job<br>2, Lost a job<br>3, Unpaid leave<br>4, Sick family member cannot work<br>99, Other<br>If OTHER, please describe: ____________________<br>-1, Refused	
G3	Have you or your household changed anything you do as a result of financial hardship related to coronavirus and associated regulations?	0, No<br>1, Yes<br>-2, Unsure<br>-1, Refused	→ G5<br><br>→ G5<br>→ G5
G4	If YES, what changed?	1, Buy less food<br>2, Leave where you were living<br>3, Take on additional jobs<br>4, Do not buy airtime<br>5, Do not pay rent<br>6, Not able to pay electricity or water bill<br>7, Not able to pay for transport for children or self<br>8, Not able to pay for car insurance<br>9, Not able to pay school fees for children or self<br>99, Other<br>If OTHER, please describe: ____________________<br>-1, Refused	
G5	Have you or anyone in your household had any changes in housing related to	0, No<br>1, Yes<br>-2, Unsure<br>-1, Refused	→ G8<br><br>→ G8<br>→ G8

Today's Date: day / month / year Study ID: ____________________

Interviewer ID: ____________________ Location/Facility: ____________________

	coronavirus and associated regulations?		
G6	If YES, what changes?	1, Left where you were living for a family or friend's home<br>2, Family members or friends moved in with you<br>3, Household members moved out<br>4, Lost housing (homeless)<br>99, Other<br>If OTHER, please describe: ______________________<br>-1, Refused	
G7	After coronavirus and associated regulations have passed, do you expect your housing to return back to the way it was or remain this way?	1, Housing changes are temporary<br>2, Housing changes are permanent<br>-2, Unsure<br>-1, Refused	
**G8 – G26: Food Insecurity Scale**			
G8	In the past four weeks, did you worry that your household would not have enough food?	0, No<br>1, Yes<br>-2, Unsure<br>-1, Refused	→ G10<br><br>→ G10<br>→ G10
G9	If YES, how often did this happen?	1, Rarely (once or twice in the past four weeks)<br>2, Sometimes (three to ten times in the past four weeks)<br>3, Often (more than ten times in the past four weeks)	
G10	In the past four weeks, were you or any household member not able to eat the kinds of foods you preferred	0, No<br>1, Yes<br>-2, Unsure<br>-1, Refused	→ G12<br><br>→ G12<br>→ G12

Today's Date: day / month / year Study ID: ____________________

Interviewer ID: ____________________ Location/Facility: ____________________

	because of a lack of resources?		
G11	If YES, how often did this happen?	1, Rarely (once or twice in the past four weeks)<br>2, Sometimes (three to ten times in the past four weeks)<br>3, Often (more than ten times in the past four weeks)	
G12	In the past four weeks, did you or any household member have to eat a limited variety of foods due to a lack of resources?	0, No<br>1, Yes<br>-2, Unsure<br>-1, Refused	→ G14<br><br>→ G14<br>→ G14
G13	If YES, how often did this happen?	1, Rarely (once or twice in the past four weeks)<br>2, Sometimes (three to ten times in the past four weeks)<br>3, Often (more than ten times in the past four weeks)	
G14	In the past four weeks, did you or any household member have to eat some foods that you really did not want to eat because of a lack of resources to obtain other types of food?	0, No<br>1, Yes<br>-2, Unsure<br>-1, Refused	→ G16<br><br>→ G16<br>→ G16
G15	If YES, how often did this happen?	1, Rarely (once or twice in the past four weeks)<br>2, Sometimes (three to ten times in the past four weeks)<br>3, Often (more than ten times in the past four weeks)	
G16	In the past four weeks, did you or any household member have to eat a smaller meal than you felt you	0, No<br>1, Yes<br>-2, Unsure<br>-1, Refused	→ G18<br><br>→ G18<br>→ G18

Today's Date: day / month / year   Study ID: ____________

Interviewer ID: ____________   Location/Facility: ____________

	needed because there was not enough food?		
G17	If YES, how often did this happen?	1, Rarely (once or twice in the past four weeks)<br>2, Sometimes (three to ten times in the past four weeks)<br>3, Often (more than ten times in the past four weeks)	
G18	In the past four weeks, did you or any household member have to eat fewer meals in a day because there was not enough food?	0, No<br>1, Yes<br>-2, Unsure<br>-1, Refused	→ G20<br><br>→ G20<br>→ G20
G19	If YES, how often did this happen?	1, Rarely (once or twice in the past four weeks)<br>2, Sometimes (three to ten times in the past four weeks)<br>3, Often (more than ten times in the past four weeks)	
G20	In the past four weeks, was there ever no food to eat of any kind in your household because of lack of resources to get food?	0, No<br>1, Yes<br>-2, Unsure<br>-1, Refused	→ G22<br><br>→ G22<br>→ G22
G21	If YES, how often did this happen?	1, Rarely (once or twice in the past four weeks)<br>2, Sometimes (three to ten times in the past four weeks)<br>3, Often (more than ten times in the past four weeks)	
G22	In the past four weeks, did you or any household member go to sleep at night hungry because there was not enough food?	0, No<br>1, Yes<br>-2, Unsure<br>-1, Refused	→ G24<br><br>→ G24<br>→ G24

Today's Date: day / month / year Study ID: ____________________

Interviewer ID: ____________________ Location/Facility: ____________________

G23	If YES, how often did this happen?	1, Rarely (once or twice in the past four weeks)<br>2, Sometimes (three to ten times in the past four weeks)<br>3, Often (more than ten times in the past four weeks)	
G24	In the past four weeks, did you or any household member go a whole day and night without eating anything because there was not enough food?	0, No<br>1, Yes<br>-2, Unsure<br>-1, Refused	→ G27<br><br>→ G27<br>→ G27
G25	If YES, how often did this happen?	1, Rarely (once or twice in the past four weeks)<br>2, Sometimes (three to ten times in the past four weeks)<br>3, Often (more than ten times in the past four weeks)	
G26	*Branching: If YES to any of G8, G10, G12, G14, G16, G18, G20, G22, G24:*<br><br>During the time of coronavirus and associated regulations, what were the reasons you did not have enough to eat?	1, Couldn't afford to buy more food<br>2, Couldn't get out to buy food (for example, didn't have transportation, or had mobility or health problems that prevented you from getting out)<br>3, Afraid to go or didn't want to go out to buy food<br>4, Couldn't get groceries or meals delivered to me<br>5, The stores didn't have the food I wanted<br>99, Other<br>If OTHER, please describe: ___________________<br>-1, Refused	
G27	Over the past four weeks, have you had access to soap or water at home for hand hygiene?	0, No<br>1, Yes<br>-1, Refused	

Today's Date: day / month / year Study ID: ________________

Interviewer ID: ________________ Location/Facility: ________________

G28	What is your primary method of transportation during coronavirus and associated regulations?	1, Walk<br>2, Public transport<br>3, Private transport<br>99, Other<br>If OTHER, please describe: ________________<br>-1, Refused	
G29	Have your methods of transportation changed due to coronavirus and associated regulations?	0, No<br>1, Yes<br>-1, Refused	→ G32<br><br>→ G32
G30	If YES, what was it before coronavirus and associated regulations?	1, Walk<br>2, Public transport<br>3, Private transport<br>99, Other<br>If OTHER, please describe: ________________<br>-1, Refused	
G31	What were your reasons for changing your method of transportation?	1, Regulations<br>2, Costs<br>3, Fear of coronavirus<br>4, Fear of crime<br>99, Other<br>If OTHER, please describe: ____________________<br>-1, Refused	
G32	Has your consumption of cigarettes changed due to coronavirus and associated regulations?	1, Yes, increased<br>2, No, stayed the same<br>3, Yes, decreased<br>4, Do not use cigarettes<br>-2, Unsure	

Today's Date: day / month / year Study ID: ____________

Interviewer ID: ____________ Location/Facility: ____________

		-1, Refused	
G33	Has your consumption of alcohol changed due to coronavirus and associated regulations?	1, Yes, increased<br>2, No, stayed the same<br>3, Yes, decreased<br>4, Do not use alcohol<br>-2, Unsure<br>-1, Refused	
G34	What level of lockdown are you currently in?	1, Level 1<br>2, Level 2<br>3, Level 3<br>4, Level 4<br>5, Level 5<br>-2, Unsure<br>-1, Refused	
G35	Did any part of your lifestyle change during the first switch from level 5 to level 4 of the lockdown?	0, No<br>1, Yes<br>-2, Unsure<br>-1, Refused	→ G37<br><br>→ G37<br>→ G37
G36	If YES, what parts of your lifestyle changed during the first switch from level 5 to level 4 of the lockdown?	1, Transportation<br>2, Food security<br>3, Housing<br>4, Hygiene (soap/water access)<br>5, Cigarette consumption<br>6, Alcohol consumption<br>7, Clinic access<br>8, Pharmacy access<br>9, Ability to pick up medications<br>10, Work/school	

Today's Date: day / month / year   Study ID: ____________________

Interviewer ID: ____________________   Location/Facility: ____________________

		99, Other<br>If OTHER, please describe: ____________________<br>-1, Refused	
G37	Did any parts of your lifestyle change during the first switch from level 4 to level 3 of lockdown?	0, No<br>1, Yes<br>-2, Unsure<br>-1, Refused	→ G39<br><br>→ G39<br>→ G39
G38	If YES, what parts of your lifestyle changed during the first switch from level 4 to level 3 of the lockdown?	1, Transportation<br>2, Food security<br>3, Housing<br>4, Hygiene (soap/water access)<br>5, Cigarette consumption<br>6, Alcohol consumption<br>7, Clinic access<br>8, Pharmacy access<br>9, Ability to pick up medications<br>10, Work/school<br>99, Other<br>If OTHER, please describe: ____________________<br>-1, Refused	
G39	Did you or your household feel prepared to resume work/school during the first switch to level 3 of the lockdown?	0, No<br>1, Yes<br>2, Not applicable (no work/school)<br>-2, Unsure<br>-1, Refused	
G40	Did you leave your home for essential activities during	0, No<br>1, Yes<br>-2, Unsure	→ G42<br><br>→ G42

Today's Date: day / month / year Study ID: ____________________

Interviewer ID: ____________________ Location/Facility: ____________________

	initial levels 4 and 5 of the national lockdown?	-1, Refused	→ G42
G41	What essential activities did you leave your home for during initial levels 4 and 5 of the national lockdown?<br>*(Check all that apply)*	1, Work<br>2, Food<br>3, Household supplies<br>4, Clinic/pharmacy<br>5, Visit friends/family<br>6, School<br>7, Take child to school<br>99, Other<br>If OTHER, please describe: ____________________<br>-1, Refused	
G42	Did you leave Umlazi during initial levels 4 and 5 of the national lockdown?	0, No<br>1, Yes<br>-2, Unsure<br>-1, Refused	→ H1<br><br>→ H1<br>→ H1
G43	What were the primary reasons you travelled outside of Umlazi?<br>*(Check all that apply)*	1, Go home to be with family<br>2, Looking for job<br>3, Could not afford to stay<br>4, Work<br>5, Visit friends/family<br>6, School<br>99, Other<br>If OTHER, please describe: ____________________<br>-1, Refused	
G44	Did you leave KZN during initial levels 4 and 5 of the national lockdown?	0, No<br>1, Yes<br>-2, Unsure	→ G47<br><br>→ G47

Today's Date: day / month / year Study ID: ____________________

Interviewer ID: ____________________ Location/Facility: ____________________

		-1, Refused	→ G47
G45	Which province(s) did you travel to?<br>*(Check all that apply)*	1, Eastern Cape<br>2, Free State<br>3, Gauteng<br>4, Limpopo<br>5, Mpumalanga<br>6, Northern Cape<br>7, North West<br>8, Western Cape<br>9, Foreign travel<br>-2, Unsure<br>-1, Refused	
G46	What were the primary reasons you travelled outside of KZN?<br>*(Check all that apply)*	1, Go home to be with family<br>2, Looking for job<br>3, Could not afford to stay<br>4, Work<br>5, Visit friends/family<br>6, School<br>99, Other<br>If OTHER, please describe: ____________________<br>-1, Refused	
G47	Do you plan to return to Durban/Umlazi?	0, No<br>1, Yes<br>-2, Unsure<br>-1, Refused	→ G51<br><br>→ G51<br>→ G51
G48	When did or do you plan to return to Durban/Umlazi?	_ _ / _ _ _ / _ _ _ _<br>(dd / mmm / yyyy)<br>[Day: 01-31; *Unknown if unknown* (pull down)	

Today's Date: day / month / year Study ID: ____________________

Interviewer ID: ____________________ Location/Facility: ____________________

		Month: Jan, Feb, Mar, Apr, May, Jun, Jul, Aug, Sep, Oct, Nov, Dec; *Unknown if unknown* (pull down)<br>Year: 2015-2023; *Unknown if unknown* (pull down)]<br>Refused: -1 for all values	
G49	*Branching: If G48 is before today's date*<br>Were you able to return?	0, No<br>1, Yes<br>-1, Refused	 → G51<br>→ G51
G50	If NO, why were you not able to return?	1, Travel restrictions<br>2, Cannot afford<br>3, Taking care of sick household member<br>99, Other<br>If OTHER, please describe: ____________________<br>-1, Refused	
G51	Did your travel affect your ability to take your HIV medication as prescribed in any way?	0, No<br>1, Yes<br>-2, Unsure<br>-1, Refused	→ H1<br><br>→ H1<br>→ H1
G52	If YES, how?	1, Unable to pick medication up<br>2, Medication ran out<br>3, Missed doses<br>4, Lost medication<br>5, Had to find a local clinic to get medication<br>6, Had to take a different medication<br>99, Other<br>If OTHER, please describe: ____________________<br>-2, Unsure<br>-1, Refused	→ H1<br>→ H1<br>→ H1<br>→ H1<br>→ H1<br><br>→ H1<br><br>→ H1<br>→ H1

Today's Date: day / month / year Study ID: ____________________

Interviewer ID: ____________________ Location/Facility: ____________________

G53	If you are taking a different medication now, what medication were you taking before?	1, TEE (also called: FDC; Atroiza; Tenofovir/Emtricitabine/Efavirenz; Atenef; Trubuss; Odimune)<br>2, TLD (Tenofovir/lamivudine/dolutegravir)<br>3, TDF/FTC/LPV/r (Tenofovir/emtricitabine/lopinavir/ritonavir; or Truvada + Aluvia)<br>4, AZT/3TC/LPV/r (Lamzed + Aluvia)<br>99, Other<br>If OTHER, please describe: ____________________<br>-2, Unsure<br>-1, Refused	
G54	What medication are you taking now?	1, TEE (also called: FDC; Atroiza; Tenofovir/Emtricitabine/Efavirenz; Atenef; Trubuss; Odimune)<br>2, TLD (Tenofovir/lamivudine/dolutegravir)<br>3, TDF/FTC/LPV/r (Tenofovir/emtricitabine/lopinavir/ritonavir; or Truvada + Aluvia)<br>4, AZT/3TC/LPV/r (Lamzed + Aluvia)<br>99, Other<br>If OTHER, please describe: ____________________<br>-2, Unsure<br>-1, Refused	
G55	Have you experienced any side-effects with your new HIV medication?	0, No<br>1, Yes<br>-2, Unsure<br>-1, Refused	→ H1<br><br>→ H1<br>→ H1
G56	If YES, what side effects? *(Check all that apply)*	1, Nausea/stomach upset<br>2, Headache	

Today's Date: day / month / year Study ID: ___________________

Interviewer ID: ___________________ Location/Facility: ___________________

		3, Dizziness<br>4, Vivid dreams<br>5, Insomnia<br>6, Depression or other mood symptoms<br>7, Rash<br>99, Other<br>If OTHER, please describe: _________________<br>-1, Refused	
**H. PHONE: Emotional Health, Depression Screening, Perceived Stress Scale**			
In this section, I will ask you some questions about your emotional wellbeing. Please answer to the best of your recollection. If you find any of the questions too personal, you do not have to answer them. However, your answers are important to us, so please answer all of the questions that you are comfortable with.			
H1	During the past month, how much of the time were you a happy person?	All of the time……………….1<br>Most of the time…………….2<br>A good bit of the time………3<br>Some of the time……………4<br>A little of the time…………...5<br>None of the time…………….6<br>Refused…………………….. -1	
H2	During the past month, how much of the time have you felt calm and peaceful?	All of the time……………….1<br>Most of the time…………….2<br>A good bit of the time………3<br>Some of the time……………4<br>A little of the time…………...5<br>None of the time…………….6<br>Refused…………………….. -1	

Today's Date: day / month / year Study ID: ____________________

Interviewer ID: ____________________ Location/Facility: ____________________

H3	During the past month, how much of the time have you been a very nervous person?	All of the time……………….1<br>Most of the time…………….2<br>A good bit of the time………3<br>Some of the time……………4<br>A little of the time…………...5<br>None of the time…………….6<br>Refused…………………….. -1	
H4	During the past month, how much of the time have you felt very sad, downhearted, or blue?	All of the time……………….1<br>Most of the time…………….2<br>A good bit of the time………3<br>Some of the time……………4<br>A little of the time…………...5<br>None of the time…………….6<br>Refused…………………….. -1	
H5	During the past month, how much of the time have you felt so sad that nothing could cheer you up?	All of the time……………….1<br>Most of the time…………….2<br>A good bit of the time………3<br>Some of the time……………4<br>A little of the time…………...5<br>None of the time…………….6<br>Refused…………………….. -1	
H6	How much of the time was your nervousness or sadness related to coronavirus?	All of the time……………….1<br>Most of the time…………….2<br>A good bit of the time………3<br>Some of the time……………4<br>A little of the time…………...5<br>None of the time…………….6<br>Refused…………………….. -1	

Today's Date: day / month / year Study ID: ____________________

Interviewer ID: ____________________ Location/Facility: ____________________

Perceived Stress Scale			
H7	In the last four weeks, how often have you felt that you were unable to control the important things in your life?	0, Never<br>1, Almost never<br>2, Sometimes<br>3, Fairly often<br>4, Very often<br>-1, Refused	
H8	In the last four weeks, how often have you felt confident about your ability to handle your personal problems?	0, Never<br>1, Almost never<br>2, Sometimes<br>3, Fairly often<br>4, Very often<br>-1, Refused	
H9	In the last four weeks, how often have you felt that things were going your way?	0, Never<br>1, Almost never<br>2, Sometimes<br>3, Fairly often<br>4, Very often<br>-1, Refused	
H10	In the last four weeks, how often have you felt difficulties were piling up so high that you could not overcome them?	0, Never<br>1, Almost never<br>2, Sometimes<br>3, Fairly often<br>4, Very often<br>-1, Refused	
H11	How much of the time was your stress related to coronavirus?	0, Never<br>1, Almost never<br>2, Sometimes	

Today's Date: day / month / year Study ID: ____________

Interviewer ID: ____________ Location/Facility: ____________

		3, Fairly often<br>4, Very often<br>-1, Refused					
**I. PHONE: Social Support**							
In this section, I will ask you some questions about social support. I will be asking you how often you feel you have support for different activities. Please answer all of the questions that you are comfortable with. On a scale from 1 to 5 where 1 is none of the time and 5 is all of the time, how often is each of the following kinds of support available to you if you need it?							
		None of the time				All of the time	Refused
I1	Someone who understands your problems	1	2	3	4	5	-1
I2	Someone to love and make you feel wanted	1	2	3	4	5	-1
I3	Someone to turn to for suggestions about how to deal with a personal problem	1	2	3	4	5	-1
I4	Someone to help you if you were confined to bed	1	2	3	4	5	-1
I5	Someone to take you to the doctor if you needed it	1	2	3	4	5	-1
I6	Someone to prepare your meals if you were unable to do it for yourself	1	2	3	4	5	-1
I7	Someone to help with daily chores if you were sick	1	2	3	4	5	-1
I8	Someone to have a good time with	1	2	3	4	5	-1
**J. Intimate Partner Violence Screening**							

Today's Date: day / month / year Study ID: ____________________

Interviewer ID: ____________________ Location/Facility: ____________________

We know that relationships can have good and bad moments. Some questions may be difficult to answer and we would like to remind you that your answers will be kept confidential. If you find any of the questions too personal, you do not have to answer them. However, your answers are important to us, so please answer all of the questions that you are comfortable with.			
J1	In the past four weeks, has your current or most recent partner punched, slapped, kicked, bit you, or caused you any type of physical harm?	0, No<br>1, Yes<br>-2, Unsure<br>-1, Refused	
J2	In the past four weeks, has your current or most recent partner insulted, ignored, or humiliated you, yelled at you, or made you feel ashamed or bad about yourself?	0, No<br>1, Yes<br>-2, Unsure<br>-1, Refused	
J3	In the past four weeks, has your current or most recent partner forced you to have sex or perform any sexual act, or touched you sexually in any way that you did not want?	0, No<br>1, Yes<br>-2, Unsure<br>-1, Refused	
J4	In the past four weeks, has your current or most recent partner made you feel afraid, unsafe, or in danger?	0, No<br>1, Yes<br>-2, Unsure<br>-1, Refused	

Today's Date: day / month / year Study ID: ____________________

Interviewer ID: ____________________ Location/Facility: ____________________

J5	Have you experienced any of the above more or less during coronavirus and the associated regulations?	1, Yes, more frequent<br>2, No change<br>3, Yes, less frequent<br>-2, Unsure<br>-1, Refused	

**K. PHONE: Qualitative Follow-Up Questions**			
The final part is a series of open-ended questions about your experiences during the COVID-19/coronavirus outbreak. Please answer all of the questions that you are comfortable with.			
K1	How much, if at all, do you think your HIV status puts you at a greater risk of getting sick from coronavirus? If so, how?		
K2	Is coronavirus impacting your access to ARV medications in any ways other than what we have discussed? If so, how?		
**L. PHONE: Reimbursement Questions**			
L1	Can we send you your incentive on this phone number, or is there another number that is better?	Same as this phone number……………………1<br>Different……………………………………..2<br>If DIFFERENT, specify:<br>__ __ __ __ __ __ __ __ __ __<br>Refused……………………………………….-1	→END
L2	Phone carrier/provider:	1, Cell-C<br>2, MTN	

Today's Date: day / month / year Study ID: ____________________

Interviewer ID: ____________________ Location/Facility: ____________________

		3, Telkom<br>4, Vodacom<br>99, OTHER<br>If OTHER, list: ________________________	
L3	Airtime voucher code:	*Text*	
