# Supplementary material for: Willingness to accept COVID-19 vaccination among people living with HIV in a high HIV prevalence community
Source: BMC Public Health. 2022 Jun 22;22:1239. doi: 10.1186/s12889-022-13623-w (PMC9214683; doi:10.1186/s12889-022-13623-w)
Supplement: Supplementary file 2 — Additional file 2. Baseline questionnaire for parent study. [file 12889_2022_13623_MOESM2_ESM.pdf]

Today's Date: day / month / year   Study ID: ____________________

Interviewer ID: ____________________   Location/Facility: ____________________

South Africa's new Central Chronic Medicine Dispensing and Distribution Program: assessing the clinic, patient and community impact

Baseline Questionnaire

**A. CONSENT SIGNATURES**			
I am glad you are interested in participating in our study. [Read consent form].			
A1	Participant signature:	______________________<br>[Signature capture]	
A2	Date of participant signature	[Autofill with today's date]	
A3	Research assistant signature:	______________________<br>[Signature capture]	
A4	Date of research assistant signature	[Autofill with today's date]	

**B. CONTACT INFORMATION**			
B1	What is your address? If you do not have a fixed address, what is the address of the place where you sleep most nights?		
	a. Street/House Number	____________ [Free text]	
	b. Suburb/Town	____________ [Free text]	
B2	Some people will be contacted after 12 months for follow-up. May we call you?	No……………………………0<br>Yes…………………………..1<br>Refused…………..……….-1	→C1<br><br>→C1
B3	What is the best phone number to call you on?	(__ __ __) __ __ __ - __ __ __ __<br>Missing: *9's throughout*	
B4	Do you have a second phone number to call you on?	No……………………………0<br>Yes…………………………..1<br>Refused…………..……….-1	→C1<br><br>→C1

Today's Date:	day / month / year	Study ID:	___________________
Interviewer ID:	___________________	Location/Facility:	___________________

B5	What is the second best phone number where we can reach you?	(__ __ __) __ __ __ - __ __ __ __<br>Missing: *9's throughout*	

**C. FAMILY OR OTHER CONTACT**			
I am going to ask you for the phone number of a family member or friend who is likely to know where you are if we are unable to contact you by phone. We will not tell this person about the study or your HIV status. We will only contact this person if we cannot reach you by phone or find you at your home.			
C1	Are you willing to provide the phone number of a friend/family member?	No……………………………0<br>Yes…………………………..1<br>Refused…………..……….-1	→D1<br><br>→D1
C2	What is your friend/family member's name? *Please type "Refused" if not given.*	____________ [Free text]	
C3	What is the best phone number where we can reach your friend/family member?	(__ __ __) __ __ __ - __ __ __ __<br>Missing: *9's throughout*	

**D. DEMOGRAPHICS**			
D1	Do you identify as male or female?	Male…………………………1<br>Female.………………………2<br>Other……..…………………3<br>Refused…………..……….-1	

Today's Date: day / month / year   Study ID: ____________

Interviewer ID: ____________   Location/Facility: ____________

D2	What is your date of birth?	_ _ / _ _ _ / _ _ _ _<br>(dd / mmm / yyyy)<br>[Day: 01-31 *Unknown if unknown/refused* (pull down)<br>Month: Jan, Feb, Mar, Apr, May, Jun, Jul, Aug, Sep, Oct, Nov, Dec; *Unknown if unknown* (pull down)<br>Year: 1920-2022; *Unknown if unknown* (pull down)]<br>*Date check – date should not be after today. Age must be at least 18.*	
D3	What is your South African ID? (13 digits)	__ __ __ __ __ __ __ __ __ __ __ __ __<br>Refused/Missing: *9's throughout*	
D4	What is your ethnic group?	Black……………………….…1<br>White…..……………………2<br>Coloured………………………3<br>Other …………………………..4<br>If OTHER, specify:______________<br>Choose not to identify………... -1	
D5	How far is your home from [Clinic]?	Less than 5 kilometers……..1<br>5 to 10 kilometers…………...2<br>10 to 30 kilometers…………3<br>More than 30 kilometers……4<br>Refused………………….. -1<br>Unknown…………………-2	

Today's Date: day / month / year   Study ID: ____________

Interviewer ID: ____________   Location/Facility: ____________

D6	How did you travel to the clinic today?	Walked……………………………………..1<br>Public transport (bus, taxi)…………………..2<br>Private transport……………………………..3<br>Other ………….……………………………..4<br>If OTHER, specify:______________<br>Refused…………………………………… -1	
D7	How long did it take you to get from your home to the clinic today?<br>(Please use leading 0's if necessary)	Time available……………………………...1<br>Refused…………………………………… -1<br>Unknown…………………………………..-2<br>__ __ hours __ __ minutes	
D8	How much money in total does it cost you to get to clinic?<br>(Please use leading 0's if necessary)	__ __ __ South African Rand<br>Refused……………………………………-1	
D9	What is the last grade of school you completed?	No school…………………...1<br>Primary……………………...2<br>Some high school..………....3<br>Matric………………………4<br>Tertiary……………………...5<br>Refused…………………… -1	
D10	Are you employed?	No……………………………0<br>Yes…………………………..1<br>Refused…………………….-1	→D12<br><br>→D12

Today's Date: day / month / year Study ID: ____________________

Interviewer ID: ____________________ Location/Facility: ____________________

D11	How many hours were you paid for work last week?	None……………………….1<br>Less than 20……………….2<br>20 to 39……………………3<br>40 or more………………...4<br>Refused…………………-1	
D12	What is your marital status?	Never married …………………….1<br>Currently married …………………2<br>Divorced/separated ……………… 3<br>Widowed ………………………….4<br>Refused…………………………….-1	 →E1
D13	Are you currently in an intimate relationship?	No…………………………………0<br>Yes, in an intimate relationship for less than 6 months …………………………. 1<br>Yes, in an intimate relationship for longer than 6 months ……………………………. 2<br>Yes, multiple intimate relationships……3<br>Refused…………………………… -1	

Today's Date:	day / month / year	Study ID:	___________________
Interviewer ID:	___________________	Location/Facility:	___________________

**E. MEDICAL HISTORY**			
In this section, I will ask you some questions about your medical history. Please answer as best as you can remember. If you find any of the questions too personal, you do not have to answer them. However, your answers are important to us, so please answer all of the questions that you are comfortable with.			
E1	Overall, how would you rate your health during the past four weeks?	Excellent……………………….…....6<br>Very good..……………....……………5<br>Good..…………………….……….…..4<br>Fair..…………………….…………3<br>Poor……..………………….....…......2<br>Very poor...…………………………...1<br>Refused………………………………-1	
E2	When were you diagnosed with HIV?	_ _ / _ _ _ / _ _ _ _<br>(dd / mmm / yyyy)<br>[Day: 01-31; *Unknown if unknown* (pull down)<br>Month: Jan, Feb, Mar, Apr, May, Jun, Jul, Aug, Sep, Oct, Nov, Dec; *Unknown if unknown* (pull down)<br>Year: 1995-2023; *Unknown if unknown* (pull down)]<br>*Date check – date should be before today*	

Today's Date:	day / month / year	Study ID:	___________________
Interviewer ID:	___________________	Location/Facility:	___________________

E3	When did you start taking medication for HIV?	_ _ / _ _ _ / _ _ _ _<br>(dd / mmm / yyyy)<br>[Day: 01-31; *Unknown if unknown* (pull down)<br>Month: Jan, Feb, Mar, Apr, May, Jun, Jul, Aug, Sep, Oct, Nov, Dec; *Unknown if unknown* (pull down)<br>Year: 1995-2023; *Unknown if unknown* (pull down)]<br>*Date check – date should be before today*	
E4	How would you rate your ability to take your prescribed HIV medication in the past month?	Very poor…………………….……1<br>Poor…………………………..2<br>Fair…………………………...3<br>Good…………………………4<br>Very good……………………5<br>Excellent……………………..6<br>Refused…………………….. -1	
E5	Many patients find it difficult to take all their HIV medications exactly as prescribed. How many doses of your HIV medication did you miss in the last 7 days?	__ __ doses<br>[Drop down with numbers 00-14; -2 and -1]<br>Don't know………………………… -2<br>Refused……………………………..-1	IF NOT FEMALE→E8
E6	Women only:<br>Do you use hormonal contraception?	No……………………………0<br>Yes…………………………..1<br>Refused……………………..-1	→E8<br><br>→E8

Today's Date: day / month / year Study ID: ____________________

Interviewer ID: ____________________ Location/Facility: ____________________

E7	What type of hormonal contraception do you use?	Injection……………………………1<br>Pill……..…………………………..2<br>Implant (implanon)……..…………3<br>Other ………………………………4<br>If OTHER, specify:________<br>Refused……………………………-1	
E8	Do you take other chronic medications?	No……………………………0<br>Yes…………………………..1<br>Refused…………………….. -1	→F1<br><br>→F1
E9	What other chronic medications do you take?	______________________<br>[*6 lines with 80 characters available per line*]	
E10	In the past 6 months have you attended a traditional healer?	No……………………………0<br>Yes…………………………..1<br>Refused…………………….. -1	
E11	In the past 6 months have you taken medicines from a traditional healer?	No……………………………0<br>Yes…………………………..1<br>Refused…………………….. -1	

Today's Date: day / month / year Study ID: ____________________

Interviewer ID: ____________________ Location/Facility: ____________________

**F. EMOTIONAL HEALTH / DEPRESSION SCREENING**			
In this section, I will ask you some questions about your emotional wellbeing. Please answer to the best of your recollection. If you find any of the questions too personal, you do not have to answer them. However, your answers are important to us, so please answer all of the questions that you are comfortable with.			
F1	During the past month, how much of the time were you a happy person?	All of the time……………….1<br>Most of the time…………….2<br>A good bit of the time………3<br>Some of the time……………4<br>A little of the time…………...5<br>None of the time…………….6<br>Refused…………………….. -1	
F2	During the past month, how much of the time have you felt calm and peaceful?	All of the time……………….1<br>Most of the time…………….2<br>A good bit of the time………3<br>Some of the time……………4<br>A little of the time…………...5<br>None of the time…………….6<br>Refused…………………….. -1	
F3	During the past month, how much of the time have you been a very nervous person?	All of the time……………….1<br>Most of the time…………….2<br>A good bit of the time………3<br>Some of the time……………4<br>A little of the time…………...5<br>None of the time…………….6<br>Refused…………………….. -1	

Today's Date:	day / month / year	Study ID:	____________________
Interviewer ID:	____________________	Location/Facility:	____________________

F4	During the past month, how much of the time have you felt very sad, downhearted, or blue?	All of the time……………….1<br>Most of the time…………….2<br>A good bit of the time………3<br>Some of the time……………4<br>A little of the time…………...5<br>None of the time…………….6<br>Refused…………………….. -1	
F5	During the past month, how much of the time have you felt so sad that nothing could cheer you up?	All of the time……………….1<br>Most of the time…………….2<br>A good bit of the time………3<br>Some of the time……………4<br>A little of the time…………...5<br>None of the time…………….6<br>Refused…………………….. -1	

G. SOCIAL SUPPORT

In this section, I will ask you some questions about social support. I will be asking you how often you feel you have support for different activities. Please answer all of the questions that you are comfortable with. On a scale from 1 to 5 where 1 is none of the time and 5 is all of the time, how often is each of the following kinds of support available to you if you need it?

		None of the time				All of the time	Refused
G1	Someone you can count on to listen to you when you need to talk	1	2	3	4	5	-1
G2	Someone to give you information to help you understand a situation	1	2	3	4	5	-1
G3	Someone to give you good advice about a crisis	1	2	3	4	5	-1

Today's Date: day / month / year Study ID: ____________________

Interviewer ID: ____________________ Location/Facility: ____________________

G4	Someone to confide in or talk to about yourself or your problems	1	2	3	4	5	-1
G5	Someone to turn to for suggestions about how to deal with a personal problem	1	2	3	4	5	-1
G6	Someone to help you if you were confined to bed	1	2	3	4	5	-1
G7	Someone to take you to the doctor if you needed it	1	2	3	4	5	-1
G8	Someone to prepare your meals if you were unable to do it for yourself	1	2	3	4	5	-1
G9	Someone to help with daily chores if you were sick	1	2	3	4	5	-1
G10	Someone who shows you love and affection	1	2	3	4	5	-1
G11	Someone to have a good time with	1	2	3	4	5	-1
G12	Someone to get together with for relaxation	1	2	3	4	5	-1
G13	Someone to do something enjoyable with	1	2	3	4	5	-1

H. SELF-EFFICACY

In this section, I will ask you some questions about your beliefs about your own abilities. Please answer all of the questions that you are comfortable with.

On a scale from 1 to 4, where 1 is not at all true and 4 is exactly true, how much do you agree with the following statements?

		Not at all true	Hardly true	Moderately true	Exactly true	Refused

Today's Date: day / month / year	Study ID: ____________________

Interviewer ID: ____________________	Location/Facility: ____________________

H1	I can always manage to solve problems if I try hard enough	1	2	3	4	-1
H2	If someone opposes me, I can find means and ways to get what I want	1	2	3	4	-1
H3	It is easy for me to stick to my aims and accomplish my goals	1	2	3	4	-1
H4	I am confident that I could deal efficiently with unexpected events	1	2	3	4	-1
H5	Thanks to my resourcefulness, I know how to handle unforeseen situations	1	2	3	4	-1
H6	I can solve most problems if I invest the necessary effort	1	2	3	4	-1
H7	I can remain calm when facing difficulties because I can rely on my coping abilities	1	2	3	4	-1
H8	When I am confronted with a problem, I can usually find several solutions	1	2	3	4	-1

Today's Date: day / month / year Study ID: ____________________

Interviewer ID: ____________________ Location/Facility: ____________________

H9	If I am in trouble, I can usually think of something to do	1	2	3	4	-1
H10	No matter what comes my way, I'm usually able to handle it	1	2	3	4	-1

I. BARRIERS AND COMPETING NEEDS

In this section, I will ask you some questions about your own barriers to getting healthcare and what competing needs you may have. Please answer all of the questions that you are comfortable with.

I am going to read you a list of things that may have been a problem when you have needed medical care. Please indicate if any of the following things have been a problem when you needed medical care <u>in the past 6 months.</u>

I1	You didn't know where to find care	No……………………………0<br>Yes…………………………..1<br>Refused…………………….. -1	
I2	You didn't think it was necessary because you didn't think you were sick enough	No……………………………0<br>Yes…………………………..1<br>Refused…………………….. -1	
I3	You couldn't get off work	No……………………………0<br>Yes…………………………..1<br>Refused…………………….. -1	
I4	You were too sick	No……………………………0<br>Yes…………………………..1<br>Refused…………………….. -1	
I5	You had to wait too long to see the nurse or doctor	No……………………………0<br>Yes…………………………..1<br>Refused…………………….. -1	

Today's Date: day / month / year Study ID: ____________________

Interviewer ID: ____________________ Location/Facility: ____________________

I6	You couldn't get to the clinic/doctor's office during the hours it was open	No……………………………0<br>Yes…………………………..1<br>Refused…………………….. -1	
I7	The nurse or doctor didn't speak your language	No……………………………0<br>Yes…………………………..1<br>Refused…………………….. -1	
I8	Transport to get to the clinic/doctor's office was a problem	No……………………………0<br>Yes…………………………..1<br>Refused…………………….. -1	
I9	You could not afford the cost of transportation	No……………………………0<br>Yes…………………………..1<br>Refused…………………….. -1	
I10	You could not afford medications	No……………………………0<br>Yes…………………………..1<br>Refused…………………….. -1	
I11	You had to take care of someone else	No……………………………0<br>Yes…………………………..1<br>Refused…………………….. -1	
I12	You were not treated with respect by the nurse or doctor	No……………………………0<br>Yes…………………………..1<br>Refused…………………….. -1	
I13	Other	No……………………………0<br>Yes…………………………..1<br>Refused…………………….. -1<br>If YES, specify:___________________	

Today's Date: day / month / year   Study ID: ____________________

Interviewer ID: ____________________   Location/Facility: ____________________

I14	In the last 6 months, have you ever had to go without health care that you needed because the money was needed for <u>food</u>?	No……………………………0<br>Yes…………………………..1<br>Refused……………………….-1	
I15	In the last 6 months, have you ever had to go without health care that you needed because the money was needed for <u>clothing</u>?	No……………………………0<br>Yes…………………………..1<br>Refused……………………………..-1	
I16	In the last 6 months, have you ever had to go without health care that you needed because the money was needed for <u>housing</u>?	No……………………………0<br>Yes…………………………..1<br>Refused…………………...……-1	
I17	In the last 6 months, have you ever had to go without health care that you needed because the money was needed for <u>anything else I haven't mentioned</u>?	No……………………………0<br>Yes…………………………..1<br>IF YES, Specify:____________<br>Refused………………………..... -1	
I18	In the last 6 months, have you ever had to go without <u>food</u> because the money was needed for health care?	No……………………………0<br>Yes…………………………..1<br>Refused……………………..…-1	
I19	In the last 6 months, have you ever had to go without <u>clothing</u> because the money was needed for health care?	No……………………………0<br>Yes…………………………..1<br>Refused……………………………..-1	

Today's Date: day / month / year	Study ID: ____________________

Interviewer ID: ____________________	Location/Facility: ____________________

I20	In the last 6 months, have you ever had to go without housing because the money was needed for health care?	No……………………………0<br>Yes…………………………..1<br>Refused………………………...……-1	
I21	In the last 6 months, have you ever had to go without anything else I haven't mentioned because the money was needed for health care?	No……………………………0<br>Yes…………………………..1<br>IF YES, Specify:____________<br>Refused…………………………..... -1	

J. HIV TREATMENT BELIEFS

In this section, I will ask you some questions about your beliefs about health, healthcare, and medicine. Please answer all of the questions that you are comfortable with.

On a scale from 1 to 5, where 1 is strongly disagree and 5 is strongly agree, how much do you agree with the following statements?

		Strongly Disagree				Strongly Agree	Refused
J1	Some traditional healers can cure HIV	1	2	3	4	5	-1
J2	Vitamins and fresh fruits and vegetables can cure HIV	1	2	3	4	5	-1
J3	Antiretroviral treatments are poisonous and make people sicker	1	2	3	4	5	-1
J4	Taking HIV medication is a good idea even if one doesn't have symptoms	1	2	3	4	5	-1
J5	HIV medications will keep me alive longer	1	2	3	4	5	-1

Today's Date:	day / month / year	Study ID:	___________________
Interviewer ID:	___________________	Location/Facility:	___________________

K. STIGMA AND HIV DISCLOSURE			
In this section, I will ask you some questions about how you feel about having HIV. We are trying to understand how patients feels about having HIV and about disclosing their HIV status. Please answer all of the questions that you are comfortable with.			
K1	It is difficult to tell people about my HIV infection	Agree…………………………...…1<br>Disagree…………………………..0<br>Refused…………………….. -1	
K2	Being HIV positive makes me feel dirty	Agree…………………………...…1<br>Disagree…………………………..0<br>Refused……………………..-1	
K3	I feel guilty that I am HIV positive	Agree…………………………...…1<br>Disagree…………………………..0<br>Refused…………………….. -1	
K4	I am ashamed that I am HIV positive	Agree…………………………...…1<br>Disagree…………………………..0<br>Refused……………………. -1	
K5	I sometimes feel worthless because I am HIV positive	Agree…………………………...…1<br>Disagree…………………………..0<br>Refused…………………….. -1	
K6	I hide my HIV status from others	Agree…………………………...…1<br>Disagree…………………………..0<br>Refused…………………….. -1	
K7	I worry that people who know I have HIV will tell others	Agree…………………………...…1<br>Disagree…………………………..0<br>Refused……………………..-1	
K8	I regret having told some people I have HIV	Agree…………………………...…1<br>Disagree…………………………..0<br>Refused……………………..-1	

Today's Date:	day / month / year	Study ID:	___________________
Interviewer ID:	___________________	Location/Facility:	___________________

K9	I worry that people may judge me when they learn I have HIV	Agree……………………………...…1<br>Disagree…………………………..0<br>Refused…………………….. -1	
K10	I work hard to keep my HIV a secret	Agree……………………………...…1<br>Disagree…………………………..0<br>Refused……………………..-1	
K11	In many areas of my life, no one knows I have HIV	Agree……………………………...…1<br>Disagree…………………………..0<br>Refused…………………….. -1	
K12	Telling someone that I have HIV is risky	Agree……………………………...…1<br>Disagree…………………………..0<br>Refused…………………….. -1	
K13	Since you were diagnosed with HIV, have you ever told anyone other than your health provider(s) you are HIV positive?	No…………………………...…0<br>Yes…………………………..1<br>Refused…………………….. -1	

L. DISCRIMINATION			
In this section, I will ask you some questions about how others have made you feel about having HIV. Please answer all of the questions that you are comfortable with.			
L1	Since you have been diagnosed, how often have you been excluded from social gatherings or activities (e.g. weddings, funerals, parties, and clubs) because of your HIV status?	Never……………………….....0<br>Once…………….……………………1<br>A few times……..…………….....2<br>Often………..…….…………..3<br>Refused…………………….. -1	

Today's Date:	day / month / year	Study ID:	___________________
Interviewer ID:	___________________	Location/Facility:	___________________

L2	How often have you been gossiped about because of your HIV status?	Never…………………………….....0<br>Once…………….….……………1<br>A few times……..….……………....2<br>Often………….…….……………..3<br>Refused…………………….. -1	
L3	How often have you been verbally insulted, harassed and/or threatened because of your HIV status?	Never…………………………….....0<br>Once…………….….……………1<br>A few times……..….……………....2<br>Often………….…….……………..3<br>Refused……………………..-1	
L4	How often have you been physically assaulted because of your HIV status?	Never…………………………….....0<br>Once…………….….……………1<br>A few times……..….……………....2<br>Often………….…….……………..3<br>Refused……………………..-1	

**M. ALCOHOL USE SCREENING**			
In this section, I will ask you some questions about your alcohol use. Please answer all of the questions that you are comfortable with. *Pull down menu for all with -1 last*			
M1	How often did you have a drink containing alcohol in the past year?	Never…………………………….....0<br>Monthly or less…..………………1<br>2-4 times a month….……………....2<br>2-3 times a week…….…………..3<br>4 or more times a week……….......4<br>Refused……………………….. -1	→N1<br><br><br><br><br>→N1

Today's Date:	day / month / year	Study ID:	___________________
Interviewer ID:	___________________	Location/Facility:	___________________

M2	How many drinks containing alcohol did you have on a typical day when you were drinking in the past year?<br>1 Beer drink = 350mL<br>1 Wine drink = 150mL<br>1 Spirit drink = 44mL	None, I do not drink……………..-3<br>1 or 2…………………………....0<br>3 or 4…………………………....1<br>5 or 6…………………………....2<br>7 to 9…………………………....3<br>10 or more……………………....4<br>Refused………………………… -1	→N1<br><br><br><br><br><br>→N1
M3	How often do you have six or more drinks on one occasion in the past year?	Never…………………………....0<br>Less than monthly………………1<br>Monthly…………….…………...2<br>Weekly…………….………….3<br>Daily or almost daily…….…......4<br>Refused………………………… -1	

**N. OTHER SUBSTANCES SCREENING**			
Now I will ask you some questions about your use of various substances. Please answer all of the questions that you are comfortable with.			
N1	How often have you smoked cigarettes?	Never ……………………....0<br>Not in the past year…....1<br>In the past year, but not in past month..2<br>In the past month…………………3<br>Refused………………………… -1	
N2	How often have you smoked Whoonga?	Never ……………………....0<br>Not in the past year…....1<br>In the past year, but not in past month..2<br>In the past month……………3<br>Refused………………………… -1	

Today's Date: day / month / year   Study ID: ____________

Interviewer ID: ____________   Location/Facility: ____________

N3	How often have you used cannabis (Dagga)?	Never ………..……………….....0<br>Not in the past year………....1<br>In the past year, but not in past month………………….……...……...2<br>In the past month………….…...………3<br>Refused………………………… -1	
N4	How often have you used ecstasy?	Never ………..……………….....0<br>Not in the past year………....1<br>In the past year, but not in past month……2<br>In the past month………….…...………3<br>Refused………………………… -1	
N5	How often have you injected drugs (example: heroin)?	Never ……………….....0<br>Not in the past year………....1<br>In the past year, but not in past month……………….……...……....2<br>In the past month………….…...………3<br>Refused………………………… -1	

Thank you very much. This completes our baseline survey. *If patient has been seen by clinic staff please continue to Post-Decision Questionnaire (PDQ).*
